# Occupational groups and risk of suicidal behavior in men: a Swedish national cohort study during 2002–2019

**DOI:** 10.1186/s12889-024-20887-x

**Published:** 2024-12-18

**Authors:** Jenny Nyberg, Catrin Wessman, Mia Söderberg, Anthony D. LaMontagne, Kjell Toren, Margda Waern, Maria Åberg

**Affiliations:** 1 Section for Clinical Neuroscience, Institute of Neuroscience and Physiology, Sahlgrenska Academy, University of Gothenburg, Medicinaregatan 11, Box 436, Gothenburg, SE-40530 Sweden; 2 Region Västra Götaland, Sahlgrenska University Hospital, Neurology Clinic, Gothenburg, Sweden; 3 Occupational and Environmental Medicine, School of Public Health and Community Medicine, Institute of Medicine, The Sahlgrenska Academy, University of Gothenburg, Gothenburg, Sweden; 4 School of Health & Social Development, Institute for Health Transformation, Deakin University, Geelong, VIC Australia; 5 Melbourne School of Population and Global Health, The University of Melbourne, Melbourne, Australia; 6 Department of Occupational and Environmental Medicine, Sahlgrenska University Hospital, Gothenburg, Sweden; 7 Department of Psychiatry and Neurochemistry, Sahlgrenska Academy, Institute of Neuroscience and Physiology, University of Gothenburg, Gothenburg, Sweden; 8 Region Västra Götaland, Sahlgrenska University Hospital, Psychosis Clinic, Gothenburg, Sweden; 9 Region Västra Götaland, Regionhälsan, Gothenburg, Sweden

**Keywords:** Epidemiology, Incidence rate ratio, Risk assessment, Self-harm, Suicide, Work-related

## Abstract

**Introduction:**

The risk of suicide has been shown to vary by occupation. We aim to identify contemporary occupational groups at greatest risk for suicidal behaviour (fatal and non-fatal), in Swedish men of working-age.

**Methods:**

A population-based cohort study of male conscripts without history of self-harm who enlisted during 1968–2001 and were followed-up during 2002–2019 (*n* = 1 542 665). Occupational groups and suicidal behaviours were identified using national registers. Incidence rate ratios (IRR) for suicidal behaviour at ages 25–65 were calculated among occupational groups, and compared to the incidence rate of the whole cohort.

**Results:**

Major occupational groups with increased risk for suicidal behavior included elementary occupations, building and manufacturing, service, care and shop sale and mechanical manufacturing and transport. Subgroup analyses revealed particularly high risks for assistant nurses, other service workers not elsewhere classified, building frame and related trades workers and cleaners and helpers.

**Conclusions:**

Men with elementary occupations as well as personal care and building and manufacturing workers were at greatest risk. This study provides a comprehensive description of risks for suicidal behaviour among occupational groups in men of working-age. These results suggest occupational groups that should be targeted for general suicide prevention intervention.

**Supplementary Information:**

The online version contains supplementary material available at 10.1186/s12889-024-20887-x.

## Introduction

Suicidal behavior i.e. suicide or non-fatal self-harm, is one of the leading causes of death and disability worldwide. Over the years, many studies have reported variations in suicide risk by occupation. However, the majority of studies are based on data gathered more than a decade ago [1–4]. Further, many only analyse occupational groups at highly aggregated levels [4–6].

In addition to suicide, non-fatal self-harm also warrants attention when assessing high-risk groups, since it is one of the strongest predictors for suicide [7]. However, little is known about the risk of non-fatal self-harm by occupation. Compared to women, mental illness including non-fatal self-harm is underdiagnosed in men, who also tend to have fewer contacts with professional mental health services [8]. This makes this group of great importance to study in terms of suicide prevention. We have previously shown that men in occupations characterized by passive work conditions (low demands and control) have a greater risk for suicidal behaviour [9], but we did not not investigate specific occupational groups.

Regarding suicide, a meta-analytical review from 2013 concluded that men in lower skilled occupations such as elementary professions, machine operators, deck crew and agricultural workers were at higher risk compared to the working-age population [1]. Men in low-skilled occupations were at highest risk of suicide in a recent British study [10] and a US report from 2016 [11]. However, a higher suicide rate has also been reported for high-skilled occupations such as veterinary surgeons [12]. High-risk occupations also appear to have changed during the years, as demonstrated by a British study comparing suicide risks between 1979–1983 and 2001–2005 [2]. Occupations with significant decreases in suicide rates over time were mainly professional or high-skilled, whereas occupations with increasing suicide rates were all low-skilled occupations.

Possible explanations for variations in results among studies may include differences in study-design, limited sample sizes, usage of different referent groups and potential confounders, as well as differences in culture and working conditions. Occupations may also have increased risk for suicidal behavior due to the underlying characteristics of the individuals employed in them [1]. However, irrespective of drivers for suicidal behaviour, it is important to identify high-risk occupational groups in men of working-age in most need of suicide prevention interventions.

Here, we utilize a large Swedish cohort (almost total national sample) of young adult men enlisting for mandatory military service between 1968 – 2001 to explore associations of occupational groups and suicidal behavior as well as non-fatal self-harm separately, during 2002–2019. The current study contributes with novel and updated results on occupation-specific risks of suicidal behaviour in men. New data are needed since previous studies lack data for the last decade and workplaces are continuously evolving. Most studies have analysed occupation-specific suicide risk, but we also present results for suicidal behavior (fatal and non-fatal) as non-fatal behaviour is a strong predictor for subsequent suicide. This study is also novel in its comprehensive analyses of occupational groups of all group-levels. Previous studies mostly focus on single occupations or occupations at highly aggregated levels. Results from this study will contribute with important insight into current risk patterns of suicidal behaviour by occupation in Sweden, identifying occupational groups at risk that should be targeted for workplace-based suicide prevention and mental health initiatives.

## Methods

### Study design

**Fig. 1:**
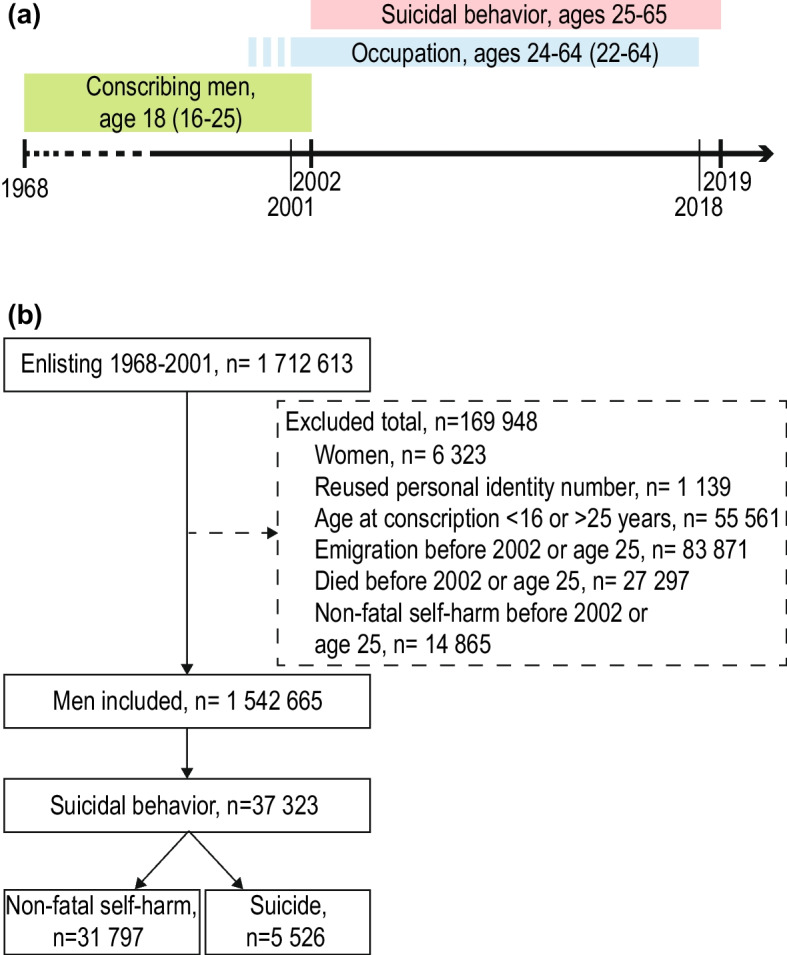
Study design and population. Schematic illustration of the study-design (**a**) where the pink line shows the period for recording suicidal behaviour and the blue line the period for recording occupation, including the three-year period where occupation was recorded for men lacking information the year prior to suicidal behaviour (blue dotted line). Flowchart of the study population (**b**) shows included and excluded men and number of men with first episode of fatal/non-fatal suicidal behaviour

This is a longitudinal population-based study of men who participated in mandatory military conscription 1968–2001 and were registered in the Swedish Military Service Conscription Register (Fig. 1a). The primary outcome (suicide or first recorded incidence of non-fatal self-harm) was identified during the follow-up from 2002 to 2019, using national registers, as was the exposure occupational group the year prior to the outcome. The follow-up period covered ages 25–65, an age group corresponding to working-age after education is completed. Linkage of register data was made possible by the unique personal identification number assigned to each registered person in Sweden. To maintain confidentiality, all data was pseudonymized and coded by Statistics Sweden after linkage.

### Study population

The source population was all men (*n* = 1 712 613) enlisting for military service 1968–2001. Men aged 16–25, with a mean age of 18.2 (SD 0.7) at enlistment, were included. Military enlistment was mandatory during this period and only men with serious disabilities or severe chronic medical or mental conditions and incarcerated individuals were granted exemption (in all, 2– 3% of the male population per year). As occupational data were not available until 2001, the observation period began in 2002 and men with intentional non-fatal self-harm (ICD-8/9: E95; ICD-10: X6, X7, X80–X84) or harm of undetermined intent (ICD-8/9: E98; ICD-10: Y1, Y2, Y30-Y34, Y87.0, Y87.2) prior to 2002 (or age 25) were excluded from the analyses. See Table 1 for details regarding relevant ICD codes. Men who died or emigrated before 2002 or before age 25 were also excluded, as were men with reused personal identity number, yielding a total of 1 542 665 men (Fig. 1b). We also performed sensitivity analyses excluding men with any psychiatric disorders or symptoms (ICD-8 and 9 codes 290–319; ICD-10 codes F00-F99) at or before conscription, yielding a 1 387 545 men.

**Table 1:** ICD-10 codes for identification of suicide (fatal) and non-fatal self-harm outcomes during follow-up

**Method**	**ICD-10**^a^	**ICD-8/9**^a,b^
All	X60–X84, Y1-Y2, Y30-Y34, Y87.0, Y87.2	E950–E959, E980-E989
Poisoning by solid or liquid substances	X60–X65, X68–X69, Y10-Y19	E950, E980
Sharp or blunt object	X78-X79	E956
Other poisoning	X66–X67, Y28-Y29	E951–E952, E981-E982
Hanging, strangulation and suffocation	X70, Y20	E953, E983
Jumping from high place	X80, Y30	E957, E987
Smoke, fire and flames	X76, Y26	
Firearms and explosives	X72–X75, Y22-Y25	E955, E985
Steam, hot vapors and hot objects	X77, Y27	
Drowning and submersion	X71, Y21	E954, E984
Jumping or lying in front of moving object	X81, Y31	
Crashing of motor vehicle	X82, Y32	
Other	X83-X84, Y33-Y34, Y87.0, Y87.2	E958–E959, E988-E989

^a^Identical ICD-codes are used for suicide and non-fatal self-harm. ICD-codes for suicide are identified in the Cause of Death Register, and non-fatal self-harm in the National Hospital Register

^b^ICD-9/8 codes were used for exclusions only

### Exposure: occupational group

Occupational group was obtained from Statistics Sweden by the annually updated Longitudinal Integration Database for Health Insurance and Labour Market Studies (Swedish acronym LISA), which integrates data from the educational and labour market sectors [13]. Occupation with the highest taxable salary each year was coded according to the Swedish Standard Classification of Occupations 1996 (SSYK96; for years 2001–2013) and 2012 (SSYK2012; for years 2014–2019). SSYK96 and SSYK2012 are Nordic adaptations of the International Standard Classification of Occupations. Occupational codes in SSYK2012 are classified from 1- to 4-digit codes, ranging from low to high specificity (1-digit level: major occupational groups; 2-digit level: sub-major occupational groups; 3-digit level: minor occupational groups and 4-digit level: unit [Statistics Sweden terminology; unit meaning unique or unitary] occupational groups). Occupational codes (at 4-digit level) in SSYK96 were subjectively translated to codes in the more updated SSYK2012. The translation was performed by two researchers independently and then compared for similarities. If the translation of a code differed, a joint decision was made after a discussion. All occupational codes from SSYK96 used in the analyses originated from these translated 4-digit codes. Hence, all codes used for the period 2001–2019 corresponded to SSYK2012-codes.

Occupational group within the year prior to the suicidal event was used as exposure. If no occupation was registered that year, we used the occupational group registered two or three years prior. If no occupation was registered during these three years, the occupational group was categorized as “Not in the labour force” and included as a separate category in the analyses. The category “Not in the labour force” included men with long-term unemployment, early retirement, sick leave or disability pension, as well as students, seasonal workers and men otherwise not in the labour force.

### Outcome: suicidal behavior

The primary outcome was suicidal behavior, a composite variable of first registered suicidal event (fatal or non-fatal) whichever came first, identified as ICD-10 codes in registers during the observation period 2002–2019 (ages 25 – 65 years). The ICD codes for both suicide (fatal self-harm) and non-fatal self-harm are the same. Suicides were identified through ICD-10 codes in the annually updated Swedish Cause of Death Register, maintained at the National Board of Health and Welfare, and cases of non-fatal self-harm were identified through ICD-10 codes in the Swedish National Hospital Register. This register includes in-patients at any hospital, out-patients in specialized medical care (including psychiatric care) and emergency department visits. ICD codes used for identification included suicide and non-fatal self-harm with clear intent (ICD-10: X6, X7, X80–X84) as well cases with undetermined intent (ICD-10: Y1, Y2, Y30-Y34, Y87.0, Y87.2) (Table 1). First events of non-fatal self-harm were also analysed separately. As the National Hospital Register does not distinguish between suicide attempts and self-harm without suicidal intent, we define non-fatal self-harm as any non-fatal self-harm behavior, regardless of the degree of suicidal intention [14].

### Other variables

#### Conscription cohorts

The included men started the study follow-up at different ages (year 2002) and therefore vary in time prior to study start to develop a career path, to receive a psychiatric diagnosis or to be excluded from the study. Moreover, possible temporal variations in rate of suicidal behaviour may exist over time. To account for this and adjust our analyses, we divided the study population into four conscription cohorts based on age year 2002. The categories were 18–27 years (reference), 28–34 years, 35–44 years and > 45 years. The age-cut offs were chosen to form equally sized categories, and correspond to the following conscription years (assumed that conscription occurred at age 18): 2002–1993 (reference), 1992–1986, 1985–1976, 1975–1968.

#### Migration and death

Information on emigration from Sweden was obtained from the LISA register and all death dates were obtained from the Swedish Cause of Death Register, which has recorded virtually all deaths in Sweden since 1961.

### Statistical analysis

Taking into account a potential violation of the dispersion assumption, a Quasi-Poisson regression was used with a unrestricted dispersion parameter [15] to analyse the incidence rate ratios (IRR), including 95% confidence intervals (CI) of suicidal behavior by occupational group, adjusted for conscription cohorts. The follow-up period began on 1st January 2002 and person-time was included until time of outcome (first episode of suicidal behaviour), death by other causes, emigration or end of follow-up on 31st December 2019, whichever occurred first. Person-time for a specific occupation was calculated as the aggregated time per occupation during the follow-up. For each occupational code, person-years, number of subjects and number of outcomes were divided among four conscription cohorts (based on age year 2002), and risks over the follow-up time were estimated. Incidence rate for the total sample (including men not in the labour force) was used as a reference. Analyses were performed separately for occupational groups on 1- to 4-digit levels. Only main effects are analysed.

Before any statistical analyses, we performed power-calculations of minimal sample sizes needed to adequately analyze an occupational group (using a calculation based on a Pearson Chi-square test for proportion difference as a conservative estimate of sample size). For the composite outcome suicidal behaviour, to be able to estimate a relative risk of 2.4, with 80% power and 5% level of significance, the power calculation indicated that an occupational group needed to include at least 17 010 person-years, where the overall reference rate was assumed to be around 80 per 100 000. The corresponding numbers for non-fatal self-harm only were inclusion of at least 22 300 person-years and assumed reference rate of 61 per 100 000. All occupational groups (2-,3- or 4-digit levels) that did not fulfill this requirement were combined and included in the analyses at a 1-digit level together with groups lacking information of the specific occupational level analysed. The combination of these groups was included as a separate exposure groups in the models, in order to correspond to a national working-age sample. We did not have statistical power to analyse suicide separately.

Sensitivity analyses of risks for suicidal behaviour were also performed excluding all men with any psychiatric disorders or symptoms at or before conscription (age 16–25 years). No corrections were made for multiple testing. All statistical calculations were performed with either SAS version 9.4 for Windows (SAS Institute, NC) or R, version 4.2.2 (R Core Team (2022).

## Results

During follow-up, 37 323 men had a record of at least one suicidal behaviour event (Fig. 1), with a mean age at event of 42.3 years. Age-group distribution at time of suicidal behaviour is shown by occupational group at a 1-digit level in Additional file 1. For 5 526 men, the first event was death by suicide; first event of non-fatal self-harm was recorded in a further 31 797 men (Table 2). The total number of suicides (with or without a record of prior non-fatal self-harm) during the follow-up time was 7 058.

**Table 2:** Crude numbers of events and events per 10 000 person-years by major occupational group 0–9 during the follow-up period 2002–2019, and mean age at start of follow-up year 2002

	**All included**			**Suicidal behaviour**		**Non-fatal self-harm**		**Suicide only**^**a**^		**All suicides**^**b**^	
**Occup** **group**	**N (%)**	**PY**	**Mean age**	**Event N**	**Event per 10 000 PY**	**Event N**	**Event per 10 000 PY**	**Event, N**	**Event per 10 000 PY**	**Event, N**	**Event per 10 000 PY**
0	10 181 (0.7)	124 361	35.5	155	12.46	136	10.94	19	1.53	23	1.85
1	80 748 (5.2)	2 283 365	40.9	1 432	6.27	1 223	5.36	209	0.92	239	1.05
2	210 523 (13.7)	4 366 179	37.1	3 363	7.70	2 836	6.50	527	1.21	642	1.47
3	200 523 (13.0)	4 117 170	36.6	3 470	8.43	3 004	7.30	466	1.13	592	1.44
4	87 420 (5.7)	1 423 213	33.4	1 695	11.91	1 437	10.10	258	1.81	340	2.39
5	142 227 (9.2)	2 476 732	31.8	4 184	16.89	3 553	14.35	631	2.55	806	3.25
6	21 160 (1.4)	561 569	38.0	861	15.33	695	12.38	166	2.96	192	3.42
7	248 566 (16.1)	4 210 461	35.2	7 354	17.47	6 357	15.10	997	2.37	1 258	2.99
8	204 280 (13.2)	3 182 728	34.9	5 174	16.26	4 396	13.81	778	2.44	970	3.05
9	65 431 (4.2)	1 078 767	32.8	2 119	19.64	1 846	17.11	273	2.53	374	3.47
NL	271 696 (17.6)	2 276 536	32.4	7 516	33.02	6 314	27.74	1 202	5.28	1 622	7.12
Total	1 542 665 (100)	26 101 081	34.9	37 323	14.30	31 797	12.18	5 526	2.12	7 058	2.70

*Abbreviations: N* number, *NL* Not in the Labour force, *Occup. Group* Occupational group, *PY* Person-Years

^a^With no prior record of non-fatal self-harm (cases included in the analyses of incidence rate ratios for suicidal behaviour)

^b^With no prior record of non-fatal self-harm

*Occupational groups:* 0 = Armed forces occupations, 1 = Managers, 2 = Occupations requiring advanced level of higher education, 3 = Occupations requiring higher education qualifications or equivalent, 4 = Administration and customer service clerks, 5 = Service, care and shop sales workers, 6 = Agricultural, horticultural, forestry and fishery workers, 7 = Building and manufacturing workers, 8 = Mechanical manufacturing and transport workers, etc., 9 = Elementary occupations

### Risk for suicidal behaviour

**Fig. 2:**
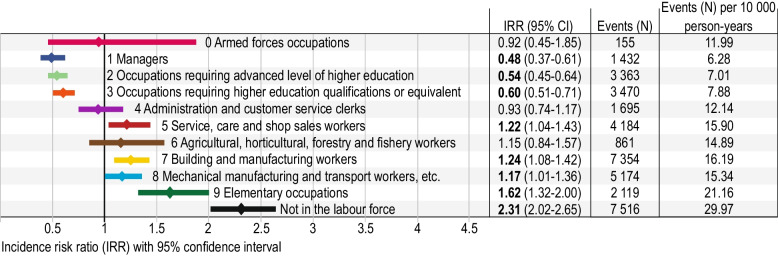
Risk for suicidal behaviour among all major occupational groups. Incidence rate ratios (IRR) and 95% confidence intervals (CI) for suicidal behaviour (fatal or non-fatal) in the major occupational groups compared to the overall incidence rate of the total study population

Apart from men without an occupational group (not in the labour force) who, as anticipated, had the highest risk for suicidal behaviour, risks were highest for *Elementary occupations*, *Building and manufacturing workers* and *Service, care and shop sales workers* (Fig. 2). *Managers* and *Occupations requiring advanced level of higher education* had the lowest risks. Risks for suicidal behaviour by major occupational groups are described in detail below, in order of highest risk. Armed forces are not described further due to low numbers. Of a total of 46 sub-major (2-digit level) occupational codes, 42 codes included sample sizes large enough to be analysed. For minor (3-digit level) occupational codes, 94 codes (of 148) included large enough sample sizes, and the corresponding figures for unit (4-digit level) occupational codes were 115 codes (of 429).

#### Elementary occupations (group 9)

**Fig. 3:**
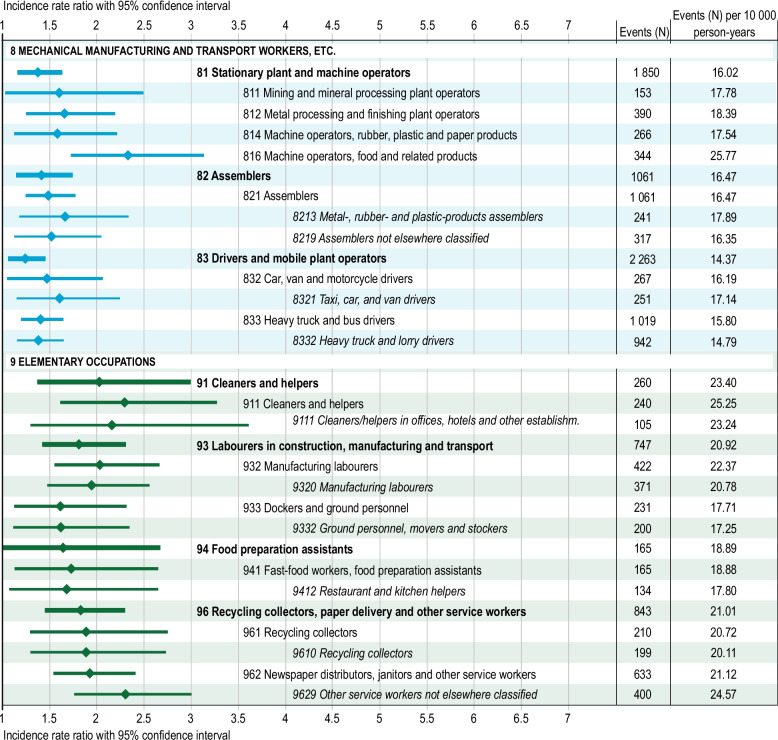
Risk for suicidal behaviour among sub-major to unite occupational groups within major occupational groups 8–9. Significant incidence rate ratios (IRR) and 95% confidence intervals (CI) for suicidal behaviour in the sub-major (2-digit level), minor (3-digit level) and unit (4-digit level) occupational groups, compared to the incidence rate of the total study population. Only occupational codes with statistically significant risk estimates and with sufficient statistical power are presented. Number of events as well as number of events per person-year for each occupational group are also presented in order to visualize sample sizes (for full data, see Additional file 1)

In general, manual workers have a higher risk than professionals (Fig. 2). Men in *Elementary occupations* had a 60% increased risk of suicidal behaviour (IRR 1.62, 95% CI 1.32–2.00) compared to the overall incidence rate of the study population. Among elementary occupations, *Cleaners and helpers* (group 91; IRR 2.02, 95% CI 1.37–3.00) and *Recycling collectors, paper delivery and other service workers* (group 96; IRR 1.82, 95% CI 1.45–2.30) had a the highest risks on a sub-major (2-digit) group level (Fig. 3 and Additional file 2). On a minor (3-digit) occupational group level, *Cleaners and helpers* (group 911; IRR 2.30, 95% CI 1.61–3.28) had increased risk as well as *Manufacturing labourers* (group 932), *Newspaper distributors, janitors and other service workers* (group 962) and *Recycling collectors* (group 961). On a unit (4-digit) occupational group level, most elementary occupations analysed were at risk, with the greatest risk for *Other service workers not elsewhere classified* (group 9629) and *Cleaners and helpers in offices, hotels and other establishments* (group 9111) (Fig. 3 and Additional file 2).

#### Building and manufacturing workers (group 7)

**Fig. 4:**
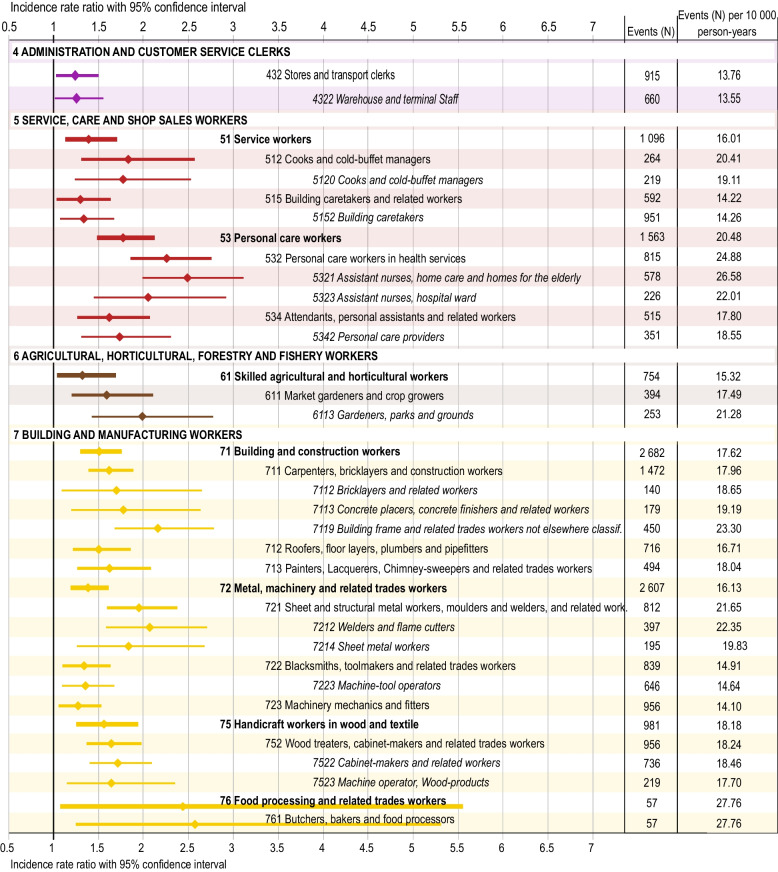
Risk for suicidal behaviour among sub-major to unit occupational groups within major occupational groups 4–7. Significant incidence rate ratios (IRR) and 95% confidence intervals (CI) for suicidal behaviour in the sub-major (2-digit level), minor (3-digit level) and unit (4-digit level) occupational groups, compared to the incidence rate of the total study population. Only occupational codes with statistically significant risk estimates and with sufficient statistical power are presented. Number of events as well as number of events per person-year for each occupational group are also presented in order to visualize sample sizes (for full data, see Additional file 1)

On a major (1-digit) occupational group level, male *Building and manufacturing workers* had the largest risk after elementary occupations, with a more than 20% increased risk of suicidal behaviour (IRR 1.24, 95% CI 1.08–1.42) (Fig. 2). *Food processing and related workers* (group 76; IRR 2.45, 95% CI 1.08–5.57) had the highest risk on a sub-major (2-digit) level, mostly explained by *Butchers, bakers and food processors* (group 761; IRR 2.58, 95% CI 1.25–5.33) (Fig. 4 and Additional file 2). However, the number of men included in this group was small (*n* = 57). Other building and manufacturing occupations at risk were *Sheet and structural metal workers, moulders and welders* (group 721; IRR 1.95, 95% CI 1.60–2.39) and *Wood treaters, cabinet-makers and related trades workers* (group 752; IRR 1.65, 95% CI 1.37–1.99).

On a unit (4-digit) occupational group level, *Building frame and related trades workers not elsewhere classified* (group 7119; IRR 2.17, 95% CI 1.68–2.79), *Welders and flame cutters* (group 7212; IRR 2.08, 95% CI 1.59–2.72) and *Sheet metal workers* (group 7214; IRR 1.84, 95% CI 1.26–2.68) had the highest risks.

#### Service, care and shop sales workers (group 5)

*Service, care and shop sale workers* had more than a 20% increased risk of suicidal behaviour (IRR 1.22, 95% CI 1.04–1.43) (Fig. 2). Within this group, *Personal care workers in health services* (group 532; IRR 2.26, 95% CI 1.85–2.76) were at greatest risk (Fig. 4 and Additional file 2). On a unit (4-digit) occupational group level, *Assistant nurses, home care and homes for the elderly* (group 5321; IRR 2.49, 95% CI 1.99–3.12), *Assistant nurses on hospital wards* (group 5323; IRR 2.05, 95% CI 1.44–2.92) and *Personal care providers* (group 5342; IRR 1.73, 95% CI 1.30–2.31) had high risks of suicidal behaviour. *Cooks and cold-buffet managers* (group 5120; IRR 1.77, 95% CI 1.23–2.53) also had an increased risk.

#### Mechanical manufacturing and transport workers, etc. (group 8)

*Mechanical manufacturing and transport workers, *etc. had a 17% increased risk of suicidal behaviour (IRR 1.17, 95% CI 1.01–1.36) (Fig. 2). On a sub-major (2-digit) level, *Stationary plant and machine operators* (group 81; IRR 1.41, 95% CI 1.17–1.70), *Assemblers* (group 82; IRR 1.43, 95% CI 1.14–1.79) and *Drivers and mobile plant operators* (group 83; IRR 2.42, 95% CI 1.77–3.30) had increased risks (Fig. 3 and Additional file 2). High risks were observed for *Machine operators, food and related products* (group 816; IRR 1.26, 95% CI 1.06–1.50) and *Car, van and motorcycle drivers* (group 832; IRR 1.50, 95% CI 1.05–2.14) on a minor (3-digit) occupational group level. Among drivers, the increased risk was explained by *Taxi, car, and van drivers* (group 8321; IRR 1.65, 95% CI 1.16–3.35).

#### Agricultural, horticultural, forestry and fishery workers (group 6)

*Agricultural, horticultural, forestry and fishery workers* did not have any increased risk for suicidal behaviour on a major (1-digit) occupational group level (Fig. 2). However, on a sub-major (2-digit) level, *Skilled agricultural and horticultural workers* had an increased risk (group 61; IRR 1.33, 95% CI 1.03–1.74) (Fig. 4 and Additional file 2). This increased risk was largely explained by the increased risk among *Gardeners, parks and grounds* (group 6113; IRR 1.95, 95% CI 1.36–2.80).

#### Administration and customer service clerks (group 4)

As a major (1-digit) occupational group, *Administration and customer service clerks* did not have elevated risk for suicidal behaviour (Fig. 2), but *Stores and transport clerks* (group 432) and *Warehouse and terminal staff* (group 4322) did (Fig. 4 and Additional file 2).

#### Occupations requiring higher education qualifications or equivalent (group 3)

As a major (1-digit) occupational group, *Occupations requiring higher education qualifications or equivalent* had a decreased risk for suicidal behaviour (IRR 0.60, 95% CI 0.51–0.71). The exception was *Social work associate professionals* who had an increased risk (group 3411; IRR 1.67, 95% CI 1.08–2.58).

#### Occupations requiring advanced level of higher education (group 2)

**Fig. 5:**
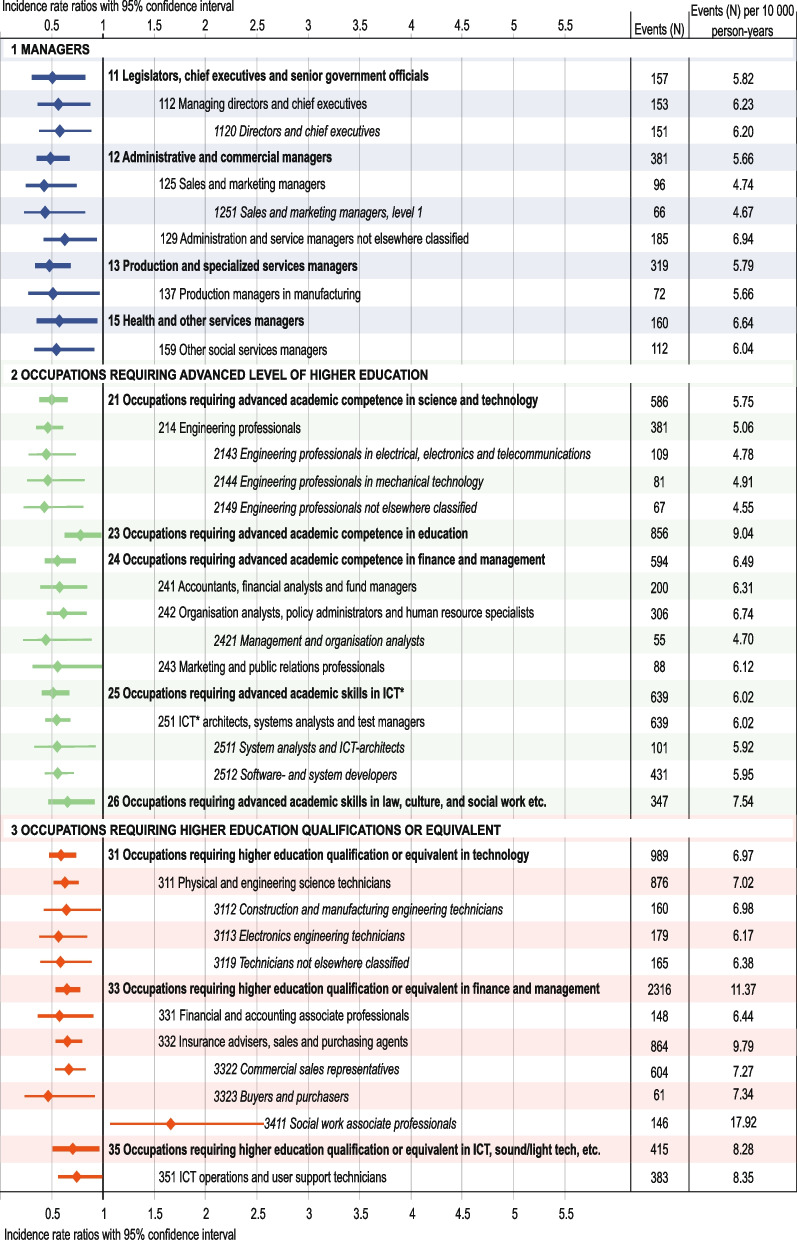
Risk for suicidal behaviour among sub-major to unit occupational groups within major occupational groups 1–3. Significant incidence rate ratios (IRR) and 95% confidence intervals (CI) for suicidal behaviour in the sub-major (2-digit level), minor (3-digit level) and unit (4-digit level) occupational groups, compared to the incidence rate of the total study population. Only occupational codes with statistically significant risk estimates and with sufficient statistical power are presented. Number of events as well as number of events per person-year for each occupational group are also presented in order to visualize sample sizes (for full data, see Additional file 1)

*Occupations requiring advanced level of higher education* had a decreased risk for suicidal behaviour (IRR 0.54, 95% CI 0.45–0.64) as a major (1-digit) occupational group (Fig. 2). Low risks were observed for *Occupations requiring advanced academic competence in science and technology* (group 21) on a sub-major (2-digit) occupational group level (Fig. 5 and Additional file 2). On a unit (4-digit) level, several occupations within engineering had a decreased risk, including *Engineering professionals not elsewhere classified* (group 2149), *Engineering professionals in electrical, electronics and telecommunications* (group 2143) and *Engineering in mechanical technology* (group 2144).

#### Managers (group 1)

Managers had the lowest risk for suicidal behaviour (IRR 0.48, 95% CI 0.37–0.61) (Fig. 2). Among managers, lowest risks were observed for *Sales and marketing managers* (group 1251). Low risks were also observed for *Production and specialized service managers* (group 13), *Administrative and commercial managers* (group 12) and *Legislators, chief executives and senior government officials* (group 11) (Fig. 5 and Additional file 2).

### Risk for suicidal behaviour in men without psychiatric disorder

We also performed sensitivity analyses of risks for suicidal behaviour in occupational groups on all four levels (1–4 digit levels) in a sub-cohort excluding men with any psychiatric disorder or symptoms at or before conscription (age 16–25 years). In general, the risk estimates were similar to when all men were included, except for men in *Elementary occupations* and *Not in the labour force* who had lower risk estimates (see Additional file 3 for results for major and sub-major occupational groups).

### Risk for non-fatal self-harm

Separate risk-analyses of the non-fatal self-harm outcome were performed for all occupational levels (Table 3). Corresponding analyses were not performed for the separate suicide outcome due to inadequate sample size.

**Table 3:** Number of events, events per 10 000 person-years, incidence rate ratios (IRR) and 95% confidence intervals (CI) for first event of non-fatal self-harm for the major occupational groups, compared to the overall incidence rate of the total study population. Only IRRs lower or higher than the incidence rate of the total study population are presented^a^

**Major occupational groups (1-digit level)**^a^	**Events** **(N)**	**Events (N) per 10 000 person-years**	**IRR**	**95% CI**
1 Managers	1 223	5.37	0.48	0.38–0.62
2 Occupations requiring advanced level of higher education	2 836	5.91	0.53	0.44–0.63
3 Occupations requiring higher education qualifications or equivalent	3 004	6.82	0.61	0.51–0.73
5 Service, care and shop sales workers	3 553	13.50	1.20	1.03–1.42
7 Building and manufacturing workers	6 357	14.00	1.25	1.09–1.44
8 Mechanical manufacturing and transport workers, etc	4 396	13.03	1.17	1.01–1.37
9 Elementary occupations	1 846	13.50	1.66	1.34–2.05
Not in the labour force	6 314	25.18	2.29	1.99–2.63
**Sub-major occupational groups (2-digit level)**^**a**^	**Events (N)**	**Events (N) per 10 000 person-years**	**IRR**	**95% CI**
11 Legislators, chief executives and senior government officials	137	5.08	0.53	0.32–0.90
12 Administrative and commercial managers	322	4.76	0.49	0.35–0.70
13 Production and specialized services managers	275	4.81	0.49	0.34–0.72
15 Health and other services managers	136	5.65	0.59	0.35–0.99
21 Occupations requiring advanced academic competence in science and technology	477	4.68	0.48	0.36–0.64
24 Occupations requiring advanced academic competence in finance and management	513	5.61	0.58	0.44–0.77
25 Occupations requiring advanced academic skills in information and communications technology	557	5.25	0.55	0.40–0.70
26 Occupations requiring advanced academic skills in law, culture, and social work etc	288	6.26	0.65	0.45–0.93
31 Occupations requiring higher education qualification or equivalent in technology	845	5.96	0.61	0.48–0.77
33 Occupations requiring higher education qualification or equivalent in finance and management	1 347	6.61	0.68	0.56–0.08
35 Occupations requiring higher education qualification or equivalent in information, communication, sound and light technologies, etc	353	7.05	0.71	0.51–0.99
51 Service workers	931	13.60	1.41	1.13–1.77
53 Personal care workers	1 320	17.29	1.77	1.46–2.15
61 Skilled agricultural and horticultural workers	621	12.61	1.33	1.03–1.74
71 Building and construction workers	2 312	15.19	1.56	1.32–1.84
72 Metal. machinery and related trades workers	2 255	13.96	1.44	1.22–1.69
75 Handicraft workers in wood and textile	843	15.62	1.61	1.28–2.03
76 Food processing and related trades workers	51	24.84	2.49	1.07–5.81
81 Stationary plant and machine operators	1 584	13.71	1.41	1.17–1.70
82 Assemblers	902	14.00	1.43	1.14–1.79
83 Drivers and mobile plant operators	1 910	12.13	1.26	1.06–1.50
91 Cleaners and helpers	231	20.79	2.13	1.42–3.21
93 Labourers in construction, manufacturing and transport	654	18.32	1.88	1.46–2.43
94 Food preparation assistants	149	17.05	1.69	1.02–2.79
96 Recycling collectors, paper delivery and other service workers	726	18.10	1.89	1.48–2.42
**Minor major occupational groups (3-digit level)**^**a**^	**Events (N)**	**Events (N) per 10 000 person-years**	**IRR**	**95% CI**
112 Managing directors and chief executives	133	5.41	0.59	0.37–0.93
125 Sales and marketing managers	83	4.09	0.43	0.24–0.77
129 Administration and service managers not elsewhere classified	151	5.66	0.61	0.39–0.94
137 Production managers in manufacturing	58	4.56	0.49	0.24–0.98
159 Other social services managers	99	5.34	0.58	0.34–0.98
214 Engineering professionals	306	4.06	0.43	0.31–0.58
221 Medical doctors	85	5.14	0.56	0.31–0.99
241 Accountants financial analysts and fund managers	173	5.45	0.58	0.39–0.88
242 Organisation analysts, policy administrators and human resource specialists	263	5.79	0.62	0.45–0.87
251 Information and communications technology architects, systems analysts and test managers	557	5.25	0.55	0.43–0.69
311 Physical and engineering science technicians	749	6.00	0.64	0.52–0.78
331 Financial and accounting associate professionals	124	5.40	0.57	0.36–0.93
332 Insurance advisers, sales and purchasing agents	748	6.36	0.67	0.55–0.83
432 Stores and transport clerks	791	11.90	1.26	1.03–1.54
512 Cooks and cold-buffet managers	237	18.32	1.88	1.32–2.67
515 Building caretakers and related workers	490	11.77	1.28	1.01–1.65
532 Personal care workers in health services	693	21.16	2.34	1.81–2.77
534 Attendants, personal assistants and related workers	428	14.80	1.58	1.21–2.05
611Market gardeners and crop growers	326	14.47	1.58	1.17–2.13
711 Carpenters, bricklayers and construction workers	1 252	15.27	1.62	1.38–1.91
712 Roofers, floor layers, plumbers and pipefitters	637	14.87	1.58	1.26–1.96
713 Painters, Lacquerers, Chimney-sweepers and related trades workers	423	15.45	1.64	1.25–2.13
721 Sheet and structural metal workers, moulders and welders, and related workers	712	18.98	2.01	1.63–2.48
722 Blacksmiths, toolmakers and related trades workers	720	12.79	1.36	1.10–1.70
723 Machinery mechanics and fitters	823	12.14	1.30	1.06–1.58
752 Wood treaters, cabinet-makers and related trades workers	825	15.72	1.67	1.38–2.03
761 Butchers, bakers and food processors	51	24.84	2.59	1.23–5.46
811 Mining and mineral processing plant operators	134	15.57	1.65	1.04–2.63
812 Metal processing and finishing plant operators	340	16.03	1.70	1.27–2.29
814 Machine operators, rubber, plastic and paper products	224	14.77	1.56	1.09–2.24
816 Machine operators, food and related products	307	23.00	2.42	1.77–3.30
821 Assemblers	902	14.00	1.48	1.22–1.78
832 Car, van and motorcycle drivers	229	13.88	1.50	1.05–2.14
833 Heavy truck and bus drivers	1 180	13.15	1.42	1.20–1.67
911 Cleaners and helpers	213	22.41	2.38	1.64–3.44
932 Manufacturing labourers	377	19.99	2.12	1.60–2.81
933 Dockers and ground personnel	193	14.80	1.57	1.07–2.31
941 Fast-food workers, food preparation assistants	149	17.05	1.75	1.13–2.72
961 Recycling collectors	182	17.95	1.93	1.30–2.88
962 Newspaper distributors, janitors and other service workers	544	18.15	1.96	1.55–2.48
**Unit major occupational groups (4-digit level)**^**a**^	**Events (N)**	**Events (N) per 10 000 person-years**	**IRR**	**95% CI**
1120 Directors and chief executives	131	5.38	0.60	0.38–0.94
1251 Sales and marketing managers, level 1	57	4.03	0.44	0.22–0.87
2143 Engineering professionals in electrical, electronics and telecommunications	81	3.55	0.39	0.22–0.68
2144 Engineering professionals in mechanical technology	70	4.25	0.40	0.25–0.86
2149 Engineering professionals not elsewhere classified	57	3.87	0.42	0.21–0.84
2421 Management and organisation analysts	46	3.93	0.43	0.20–0.92
2511 System analysts and ICT-architects	86	5.04	0.55	0.32–0.96
2512 Software- and system developers	375	5.17	0.56	0.43–0.73
3113 Electronics engineering technicians	149	5.14	0.56	0.37–0.86
3119 Technicians not elsewhere classified	136	5.26	0.58	0.37–0.94
3322 Commercial sales representatives	525	6.32	0.69	0.55–0.87
3323 Buyers and purchasers	53	4.42	0.49	0.24–0.99
3411 Social work associate professionals	126	15.47	1.70	1.07–2.69
4322 Warehouse and terminal Staff	575	11.80	1.29	1.04–1.61
5120 Cooks and cold-buffet managers	196	17.11	1.82	1.25–2.63
5152 Building caretakers	489	11.80	1.33	1.05–1.69
5321 Assistant nurses, home care and homes for the elderly	490	22.53	2.46	1.94–3.13
5323 Assistant nurses, hospital ward	194	18.89	2.08	1.43–3.02
5342 Personal care providers	303	16.01	1.77	1.31–2.39
6113 Gardeners, parks and grounds	207	17.41	1.95	1.36–2.80
7112 Bricklayers and related workers	125	16.65	1.76	1.11–2.80
7113 Concrete placers, concrete finishers and related workers	154	16.51	1.81	1.20–2.75
7119 Building frame and related trades workers not elsewhere classified	378	19.57	2.15	1.64–2.82
7125 Plumbing and central heating fitters	268	12.94	1.42	1.03–1.95
7212 Welders and flame cutters	349	19.65	2.15	1.63–2.85
7214 Sheet metal workers	168	17.09	1.87	1.26–2.80
7223 Machine-tool operators	559	12.67	1.39	1.11–1.74
7522 Cabinet-makers and related workers	648	16.25	1.79	1.45–2.21
7523 Machine operator, Wood-products	176	14.22	1.57	1.06–2.32
8213 Metal-, rubber- and plastic-products assemblers	211	15.64	1.71	1.20–2.45
8219 Assemblers not elsewhere classified	266	13.72	1.50	1.09–2.07
8321 Taxi, car, and van drivers	216	14.75	1.65	1.16–3.35
8332 Heavy truck and lorry drivers	810	12.72	1.41	1.17–1.71
9111 Cleaners and helpers in offices, hotels and other establishments	92	20.37	2.19	1.28–3.76
9320 Manufacturing labourers	332	18.60	2.05	1.54–2.73
9332 Ground personnel, movers and stockers	170	14.67	1.61	1.08–2.40
9412 Restaurant and kitchen helpers	121	16.07	1.72	1.07–2.75
9610 Recycling collectors	174	17.58	1.96	1.32–2.90
9629 Other service workers not elsewhere classified	349	21.44	2.39	1.81–3.16

*Abbreviations: CI* Confidence Interval, *IRR* Incidence Rate Ratio

^a^All risk estimates are not presented in the table due to space (for non-significant results, please see Additional file 4)

In general, risk estimates for non-fatal self-harm paralleled estimates for the composite outcome. High risks for non-fatal self-harm were found for occupations within personal care workers, in particularly in health services such as *Assistant nurses in home care and homes for the elderly* (group 5321) and *Assistant nurses, hospital ward* (group 5323). Elementary occupations also had an increased risk including *Other service workers not elsewhere classified* (group 9629), *Cleaners and helpers in offices, hotels and other establishments* (group 9111) and *Manufacturing labourers* (group 9320). Several occupations within building and manufacturing had high risk for non-fatal self-harm such as *Building frame and related trade workers not elsewhere classified* (group 7119), *Welders and flame cutters classified* (group 7212) and *Sheet metal workers* (group 7212). Although many occupations had increased risks (see Table 3 and Additional file 4 for full data), it should be noted that a 2.5-fold risk was also observed in the group *Food processing and related trades workers* (group 76) which was driven by *Food processing and related trades workers* (761). However, these groups contained few numbers (*n* = 57) which resulted in large CIs (Fig. 4). Non-significant risk estimates for non-fatal self-harm can be found in Additional file 4.

### Crude number of events of suicidal behavior, non-fatal self-harm and suicide per 10 000 person-years

Men without an occupational group (not in the labour force) had the highest crude number of both suicidal behavior events and events of non-fatal self-harm per 10 000 person-years. Among the major occupational groups, *Elementary occupations*, *Building and manufacturing workers* and *Service, care and shop sales workers* had the highest crude number of both suicidal behavior events and events of non-fatal self-harm per 10 000 person-years (Table 2). For suicide events, *Elementary occupations*, *Agricultural, horticultural, forestry and fishery workers* and *Service, care and shop sales workers* had the highest crude number per 10 000 person-years.

## Discussion

### Main findings

This study showed the greatest risks for suicidal behaviour in elementary occupations, building and manufacturing workers, service, personal care workers and mechanical manufacturing and transport workers. Other occupations with an increased risk were gardeners (parks and grounds), social work associate professionals and taxi, car, and van drivers. Separate risk estimates for non-fatal self-harm paralleled estimates for suicidal behaviour. Men not in the labour force had the overall highest risk for suicidal behaviour.

### Comparison with other studies

Although there are studies on risks for suicide in different occupations from Australia [5], Switzerland [6], U.S. [16] and England [10], studies including non-fatal self-harm as an outcome are scarce.

We found that basically all analysed groups within elementary occupations had an increased risk for suicidal behaviour. Among these, the risks were highest for other service workers not elsewhere classified and cleaners and helpers. Risks were also elevated for manufacturing labourers, recycling collectors, fast-food workers and dockers and ground personnel. An increased risk for suicide in elementary occupations has been reported in some studies [1, 5, 10], but not in others [4, 6]. In a Swedish study, the risk for suicide was not increased for elementary occupations, but there were fewer suicide cases, a shorter follow-up time and data were collected over a decade ago (2006–2010) [4]. A higher risk for suicidal behaviour in low-skilled occupations might partly be explained by a higher level of psychosocial job stressors. Men working in occupations with low control and high strain do have an increased risk for suicide and non-fatal self-harm both internationally [17] and in Sweden [9]. The increased risk might also partly be explained by socio-economic forces, reflecting lower income and education level as well as poorer general health [1, 2]. Socio-economic factors have been suggested to be a stronger determinant for occupational patters of suicide risk for men than for women [2]. A Lower risk estimates in analyses excluding men with prior psychiatric disorders may indicate a selection of more vulnerable men into elementary occupations.

An increased risk was also observed for several building and manufacturing occupations, a group lately having received attention for an increased suicide rate, especially among lower-skilled workers [1, 4, 10, 16, 18, 19]. Analyses excluding men with prior psychiatric disorders did not indicate a selection of vulnerable individuals into this occupational group. According to the U.S. Centers for Disease Control and Prevention, male workers within construction and extraction had the highest suicide rate of all occupational categories in 2012 and again in 2015, with a 22% increase between these years [16]. Several contributors to increased suicide risk among construction workers have been considered, including job insecurity, transient working conditions, workplace injury and fear of legal prosecution in relation to debt and conduct at work [20]. In Australia, the high suicide rate among construction workers led to the implementation of a multi-modal suicide prevention program MATES in Construction [21], and the rate is now decreasing [19].

We also observed a high risk for suicidal behaviour in personal care workers, particularly evident among assistant nurses and personal care providers. Risk estimates for suicidal behavior among personal care workers were slightly attenuated in analyses excluding men with prior psychiatric disorders. This may be indicative of a selection of vulnerable men, as also suggested for male nursing/health care students by a resent Swedish study [22]. A higher suicide risk for male nurses has been reported previously [4, 23, 24]. A combination of factors may contribute to the increased risk among male nurses including psychiatric illness, work-related stressors and also gender norms and stigma [24].

Social work associate professionals also had an increased risk, paralleling observations regarding male welfare support workers in an Australia [23]. Social care workers constitute an occupational group with many psychological stressors, increased exposure to traumatic events at work, high degree of job insecurity and limited access to organisational support [23, 25]. Increased risks for mood disorder, stress-related disorders and antidepressant use have been previously reported for male social care workers [25, 26]. Providing services to vulnerable populations may lead to emotional exhaustion, secondary traumatic stress and compassion fatigue [26]. These are all factors known to increase the risk of suicide mortality in general [27, 28]. Personality traits may influence occupational choices [29] as well as vulnerability for mood disorders and suicidal ideation [30], and men who choose to work within social care might be more susceptible to mental illness to begin with [25].

Workers within mechanical manufacturing and transport services were also at risk, a risk estimate mostly driven by machine operators, assemblers and taxi-, car-and van-drivers. Our results regarding transport workers are in line with a recent systematic review stating that this group has a significantly higher risk for suicide compared to the general working population [31]. The transport sector may have one of the highest levels of work stressors [31–33], including physical work factors such as environmental overstimulation, difficult ergonomic positions and adverse temperatures [27]. Stressors also include psychosocial risk factors with a reduced capacity for decision-making and control in combination with high demands, high degree of time pressure, irregular shifts with insufficient rest periods and a high level of responsibility for traffic safety [27, 31–33].

Gardeners (parks and grounds) also had an increased risk, paralleling the increased suicide risk reported for male gardeners in England [10]. These occupations may be regarded as elementary and reflect similar explanatory factors for the increased risk.

To our knowledge, no previous studies have analysed occupational risk for non-fatal suicide behavior as well as a combination of fatal and non-fatal outcomes. Separate analyses of non-fatal self-harm yielded risk estimates similar to those for the composite outcome (any suicidal behaviour). Further research is required to understand the drivers of the elevated risks for suicidal behaviour by occupational group. General suicide prevention programs should prioritise identified high risk groups, and such programs could be further tailored to the particular work- and non-work-related risk and protective factors for each group.

### Strengths and imitations

This study presents novel data with strengths including a large sample size (almost 1.5 million men) and a prospective, population-based design with updated data obtained from high-quality national registers. It is the most in-depth study in a Swedish setting thus far, involving a follow-up time up to 2019 and analyses on all four occupational levels. Moreover, we provide novel separate risk estimates for non-fatal self-harm.

This study also has limitations. As a reference in the analyses, we used the incidence rate for suicidal behaviour in our whole sample, including men not in the labour force. The rationale for this is that we aimed at a reference population as similar to a national sample of working-age men as possible. Including men not in the labour force in the reference would however give a higher incidence rate for the reference and thus yield more conservative risk estimates.

Incidence of non-fatal self-harm may be underestimated since some men do not seek care in connection with self-harm, and others might have primary care contact only. Neither of these situations can be identified in the national registers. As data from the National Hospital Register includes deliberate self-harm both with and without suicidal intent, we were not able to distinguish between these two. However, self-harm without suicidal intent is less prevalent in midlife than in young adulthood [34] and we do not expect that this is a major issue. Another consideration is that men were censored at their first self-harm event, and it is likely that some men with an initial non-fatal episode died later by suicide.

Rates of suicidal behaviour may fluctuate over time and also vary with age. Men entered the current study at different ages (year 2002), and therefore differ in time prior to study start to already have experienced mental illness, unemployment, relational difficulties or other known risk factors for suicidal behavior [35], which is why we adjusted for conscription cohorts. However, we did not adjust for age at event, which may influence our results.

We were not able to perform separate risk-analyses for suicide deaths due to limited cohort size. However, we have included crude numbers of events per 10 000 person-years. Although we did exclude men with previous non-fatal self-harm prior to start of follow-up and performed sensitivity analyses excluding men with psychiatric disorders at young adulthood, residual and unmeasurable confounding may still be present.

Our results pertain to men only and cannot be directly extrapolated to women. This is an important limitation given that occupations often vary with gender, as do types of suicidal behavior. Occupational group was not measured longitudinally and possible effects of individual changes in occupation are unknown. However, we assume that the impact is probably small.

## Conclusions

Overall, risk estimates for suicidal behaviour (both fatal and non-fatal) were greatest in elementary occupations service followed by building and manufacturing workers, personal care and shop sales workers and mechanical manufacturing and transport workers, and the same patterns were seen for behaviors with non-fatal outcomes. This study provides a comprehensive description of occupational groups at risk for suicidal behaviour among working age men. Occupational groups at risk should be prioritised/targeted for workplace-based suicide prevention and mental health initiatives. Future research should focus on investigating drivers for the associations between occupation and risk for suicidal behaviour to gain more knowledge and to design occupation-specific suicide prevention initiatives.

## Supplementary Information

Additional file 1. Age-group at time of suicidal behaviour by occupational group at a 1-digit level (major occupational group). Percent of first-time suicidal behaviour within a major occupational group occurring within specified age-groups (18–27 years, 28–34 years, 35–55 years and 45–65 years)

Additional file 2. Risk for suicidal behaviour among occupational groups. Description: Number of events, events per 10 000 person-years, incidence rate ratios (IRR) and 95% confidence intervals (CI) for suicidal behaviour calculated separately for 1- to 4-digit level occupational groups, compared to the incidence rate of the total study population. Only groups with sufficient sample size are shown

Additional file 3. Risk for suicidal behaviour among men without psychiatric disorders at or before conscription. Incidence rate ratios (IRR) with 95% confidence intervals for suicidal behaviour (fatal or non-fatal) in analyses where men with psychiatric disorders at conscription (age 16–25) were excluded (light grey bars), or not (dark grey bars). In (a) IRRs lower or higher than the incidence rate of the total study population for the major occupational groups (1-digit level) are presented In (a) and IRRs higher than the incidence rate of the total study population for the sub-major occupational groups (2-digit level) are shown

Additional file 4. Non-significant risk estimates for non-fatal self-harm among occupational groups. Number of events, events per 10 000 person-years, incidence rate ratios (IRR) and 95% confidence intervals (CI) for non-fatal self-harm calculated separately for 1- to 4-digit level occupational groups compared to the incidence rate of the total study population. Only groups with sufficient sample size are shown

## Data Availability

Raw data generated for the current study is not publicly available for ethical reasons but data on group level are available from the corresponding author on reasonable request. To obtain raw data, researchers can follow the Swedish ethical review procedures for research on the same study population (https://etikprovningsmyndigheten.se/en/). For advice on how to obtain access to data, contact the Head of Department at the Institute of Medicine, University of Gothenburg (Jan Boren, jan.boren@wlab.gu.se).

## Abbreviations

SD: Standard Deviation
ICD: The International Classification of Diseases
SSYK: The Swedish Standard Classification of Occupations
LISA: Longitudinal Integration Database for Health Insurance and Labour Market Studies
IRR: Incidence Rate Ratio
CI: Confidence Interval
