# Supplementary material for: Occupational groups and risk of suicidal behavior in men: a Swedish national cohort study during 2002–2019
Source: BMC Public Health. 2024 Dec 18;24:3515. doi: 10.1186/s12889-024-20887-x (PMC11657517; doi:10.1186/s12889-024-20887-x)
Supplement: Supplementary file 1 — Additional file 1. Age-group at time of suicidal behaviour by occupational group at a 1-digit level (major occupational group). Percent of first-time suicidal behaviour within a major occupational group occurring within specified age-groups (18–27 years, 28–34 years, 35–55 years and 45–65 years) [file 12889_2024_20887_MOESM1_ESM.pdf]

Additional file 1 Nyberg et al., 2024. Occupational groups and risk of suicidal behavior in men: a Swedish national cohort study during 2002-2019.

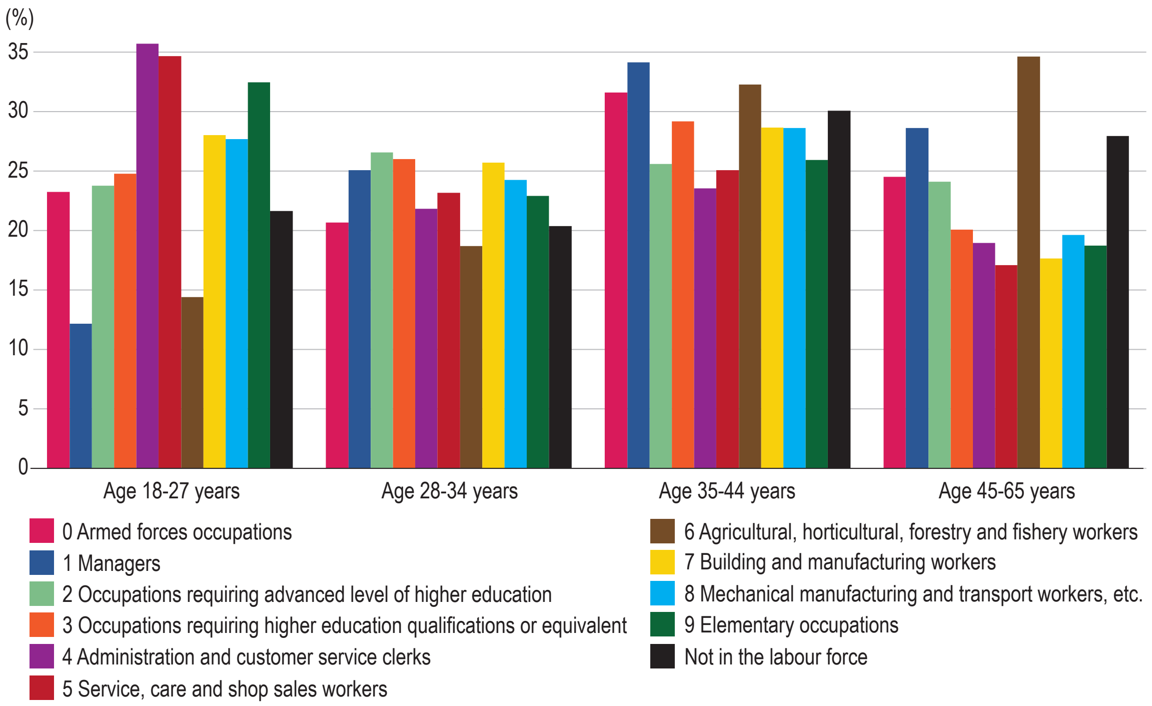

Additional file 1. Age-group at time of suicidal behaviour by occupational group at a 1-digit level (major occupational group).

Percent of first-time suicidal behaviour within a major occupational group occurring within specified age-groups (18-27 years, 28-34 years, 35-55 years and 45-65 years).
