# Supplementary material for: Occupational groups and risk of suicidal behavior in men: a Swedish national cohort study during 2002–2019
Source: BMC Public Health. 2024 Dec 18;24:3515. doi: 10.1186/s12889-024-20887-x (PMC11657517; doi:10.1186/s12889-024-20887-x)
Supplement: Supplementary file 2 — Additional file 2. Risk for suicidal behaviour among occupational groups. Description: Number of events, events per 10 000 person-years, incidence rate ratios (IRR) and 95% confidence intervals (CI) for suicidal behaviour calculated separately for 1- to 4-digit level occupational groups, compared to the incidence rate of the total study population. Only groups with sufficient sample size are shown [file 12889_2024_20887_MOESM2_ESM.pdf]

Additional file 2. Risk for suicidal behaviour among occupational groups. Number of events, events per 10 000 person-years, incidence rate ratios (IRR) and 95% confidence intervals (CI) for suicidal behaviour calculated separately for 1- to 4-digit level occupational groups, compared to the incidence rate of the total study population. Only groups with sufficient sample size are shown.

**Sub-major occupational groups** (2-digit level)	**Events (N)**	**Events (N) per 10 000 person-years**	**IRR**	**95% CI**
**11 Legislators, chief executives and senior government officials**	157	5.82	**0.50**	**0.31-0.83**
**12 Administrative and commercial managers**	381	5.66	**0.49**	**0.35-0.68**
**13 Production and specialized services managers**	319	5.79	**0.48**	**0.34-0.68**
14 Education managers	16	7.79	0.73	0.16-3.40
**15 Health and other services managers**	160	6.64	**0.57**	**0.35-0.94**
16 Financial and insurance services branch managers	67	8.18	0.73	0.33-1.50
17 Hotel, restaurant, retail and other services managers	311	8.38	0.72	0.50-1.04
1 Combined data for managers lacking 2-digit information or power	*21*	*4.31*	*0.37*	*0.10-1.40*
**21 Occupations requiring advanced academic competence in science and technology**	586	5.75	**0.49**	**0.38-0.65**
22 Occupations requiring advanced academic competence in health care	341	8.54	0.74	0.53-1.05
**23 Occupations requiring advanced academic competence in education**	856	9.04	**0.78**	**0.62-0.98**
**24 Occupations requiring advanced academic competence in finance and management**	594	6.49	**0.56**	**0.43-0.73**
**25 Occupations requiring advanced academic skills in information and communications technology (ICT)**	639	6.02	**0.52**	**0.40-0.67**
**26 Occupations requiring advanced academic skills in law, culture, and social work etc.**	347	7.54	**0.65**	**0.46-0.92**
**31 Occupations requiring higher education qualification or equivalent in technology**	989	6.97	**0.60**	**0.48-0.75**
32 Occupations requiring higher education qualification or the equivalent in healthcare and laboratory	85	9.26	0.80	0.41-1.57
**33 Occupations requiring higher education qualification or equivalent in finance and management**	2 316	11.37	**0.65**	**0.55-0.78**
34 Occupations requiring higher education qualification or the equivalent in culture, wellness and social work	434	12.19	1.05	0.77-1.43
**35 Occupations requiring higher education qualification or equivalent in information, communication (ICT), sound and light technologies, etc.**	415	8.28	**0.71**	**0.52-0.97**
41 Office clerks	440	10.05	0.87	0.64-1.18
42 Customer services clerk	130	12.08	1.03	0.60-1.79
43 Stock clerks	915	13.76	1.19	0.95-1.49
44 Other office and service workers	192	11.31	0.99	0.63-1.55
4 Combined data for administration and customer service clerks lacking 2-digit information or power	*18*	*11.09*	*1.03*	*0.24-4.43*
**51 Service workers**	1 096	16.01	**1.39**	**1.13-1.71**
52 Sales workers	1 123	12.74	1.10	0.89-1.35
**53 Personal care workers**	1 563	20.48	**1.77**	**1.48-2.12**
54 Surveillance workers and fire fighters	402	30.30	1.15	0.83-1.58
**61 Skilled agricultural and horticultural workers**	754	15.32	**1.33**	**1.05-1.70**
62 Skilled forestry and fishery workers	70	12.20	1.07	0.51-2.26
6 Combined data for agricultural, horticultural, forestry and fishery workers lacking 2-digit information or power	*37*	*13.05*	*1.11*	*0.40-3.07*
**71 Building and construction workers**	2 682	17.62	**1.51**	**1.30-1.76**
**72 Metal, machinery and related trades workers**	2 607	16.13	**1.39**	**1.19-1.62**
73 Precision-instrument makers, printing and handicraft workers	220	12.53	1.08	0.71-1.65
74 Electrical and electronic trades workers	693	11.06	0.95	0.74-1.23
**75 Handicraft workers in wood and textile**	981	18.18	**1.57**	**1.26-1.95**
**76 Food processing and related trades workers**	57	27.76	**2.45**	**1.08-5.57**
7 Combined data for building and manufacturing workers lacking 2-digit information or power	*114*	*27.32*	***2.32***	***1.29-4.16***
**81 Stationary plant and machine operators**	1 850	16.02	**1.38**	**1.16-1.64**
**82 Assemblers**	1 061	16.47	**1.42**	**1.15-1.75**
**83 Drivers and mobile plant operators**	2 263	14.37	**1.24**	**1.06-1.46**
**91 Cleaners and helpers**	260	23.40	**2.02**	**1.37-3.00**
**93 Labourers in construction, manufacturing and transport**	747	20.92	**1.81**	**1.42-2.31**
**94 Food preparation assistants**	165	18.88	**1.64**	**1.01-2.67**
**96 Recycling collectors, paper delivery and other service workers**	843	21.01	**1.82**	**1.45-2.30**

9 Combined data for elementary occupations lacking 2-digit information or power	*104*	*23.17*	***2.00***	***1.09-3.69***
**Not in the labour force**	7 516	29.97	**2.61**	**2.32-2.93**

**Minor occupational groups** (3-digit level)	**Events (N)**	**Events (N) per 10 000 person-years**	**IRR**	**95% CI**
011 Commissioned armed forces officers	149	12.38	1.12	0.72-1.76
0 Combined data for armed forces occupations lacking 3-digit information or power	*6*	*6.75*	*0.61*	*0.07-5.63*
**112 Managing directors and chief executives**	153	6.23	**0.56**	**0.36-0.88**
121 Finance managers	45	5.24	0.47	0.21-1.06
123 Administration and planning managers	19	4.83	0.44	0.13-1.54
**125 Sales and marketing managers**	96	4.74	**0.42**	**0.24-0.74**
**129 Administration and service managers not elsewhere classified**	185	6.94	**0.63**	**0.42-0.94**
131 Information and communications technology service managers	26	5.71	0.50	0.17-1.44
132 Supply, logistics and transport managers	20	4.41	0.38	0.11-1.29
136 Production managers in construction and mining	114	7.01	0.63	0.38-1.05
**137 Production managers in manufacturing**	72	5.66	**0.51**	**0.27-0.97**
141 Primary and secondary schools and adult education managers	15	7.73	0.75	0.19-3.07
**159 Other social services managers**	112	6.04	**0.55**	**0.33-0.92**
161 Financial and insurance managers	67	8.18	0.74	0.38-1.44
171 Hotel and conference managers	34	8.50	0.74	0.29-1.88
172 Restaurant managers	176	9.66	0.87	0.58-1.32
179 Other services managers not elsewhere classified	52	7.46	0.67	0.32-1.43
1 Combined data for managers lacking 3-digit information or power	*246*	*5.14*	***0.47***	***0.33-0.67***
**214 Engineering professionals**	381	5.06	**0.46**	**0.34-0.61**
217 Designers	33	6.94	0.63	0.24-1.61
221 Medical doctors	117	7.08	0.65	0.39-1.07
222 Nursing professionals	47	11.19	1.01	0.46-2.23
231 University and higher education teachers	145	7.62	0.69	0.44-1.09
232 Vocational education teachers	64	12.06	1.11	0.56-2.20
233 Secondary education teachers	106	7.45	0.68	0.40-1.55
234 Primary- and pre-school teachers	433	10.03	0.91	0.70-1.19
235 Teaching professionals not elsewhere classified	94	8.99	0.82	0.47-1.44
**241 Accountants, financial analysts and fund managers**	200	6.31	**0.57**	**0.39-0.84**
**242 Organisation analysts, policy administrators and human resource specialists**	306	6.74	**0.61**	**0.45-0.84**
**243 Marketing and public relations professionals**	88	6.12	**0.55**	**0.31-0.99**
**251 ICT architects, systems analysts and test managers**	631	6.02	**0.54**	**0.43-0.68**
261 Legal professionals	70	6.18	0.56	0.29-1.08
264 Authors, journalists and linguists	97	7.11	0.64	0.37-1.12
265 Creative and performing artists	76	8.48	0.77	0.41-1.43
2 Combined data for occupations requiring advanced level of higher education lacking 3-digit information or power	*467*	*8.40*	***0.76***	***0.59-0.99***
**311 Physical and engineering science technicians**	876	7.02	**0.63**	**0.52-0.77**
312 Construction and manufacturing supervisors	51	6.46	0.57	0.27-1.23
315 Ship and aircraft controllers and technicians	49	7.91	0.71	0.33-1.55
321 Medical and pharmaceutical technicians	80	9.14	0.83	0.45-1.53
**331 Financial and accounting associate professionals**	148	6.44	**0.58**	**0.37-0.92**
**332 Insurance advisers, sales and purchasing agents**	864	9.79	**0.66**	**0.55-0.80**
333 Business services agents	261	8.33	0.75	0.54-1.06
335 Tax and related government associate professionals	121	8.05	0.73	0.45-1.21
336 Police officers	148	9.63	0.88	0.56-1.38
341 Social work and religious associate professionals	158	16.55	1.50	0.97-2.32
342 Athletes, fitness instructors and recreational workers	108	11.20	1.00	0.59-1.70
343 Photographers, interior decorators and entertainers	72	9.69	0.86	0.45-1.64
351 ICT operations and user support technicians	383	8.35	0.75	0.57-1.00
3 Combined data for occupations requiring higher education qualifications or equivalent lacking 3-digit information or power	*151*	*8.42*	*0.78*	*0.50-1.22*
411 Office assistants and other secretaries			0.91	0.70-1.19
422 Client information clerks			1.10	0.67-1.81
**432 Stores and transport clerks**	915	13.76	**1.25**	**1.03-1.51**
442 Postmen and postal facility workers	122	12.26	1.07	0.71-1.61
4 Combined data for administration and customer service clerks lacking 3-digit information or power	*39*	*9.61*	*0.90*	*0.38-2.15*
**512 Cooks and cold-buffet managers**	264	20.41	**1.83**	**1.30-2.57**

513 Waiters and bartenders	50	15.39	1.43	0.66-3.10
**515 Building caretakers and related workers**	592	14.22	**1.30**	**1.03-1.63**
522 Shop staff	984	12.36	1.12	0.93-1.35
523 Cashiers and related clerks	24	12.30	1.15	0.38-3.48
524 Event seller and telemarketers	52	22.19	2.07	0.97-4.41
531 Child care workers and teachers aides	171	15.02	1.35	0.89-2.06
**532 Personal care workers in health services**	815	24.88	**2.26**	**1.85-2.76**
**534 Attendants, personal assistants and related workers**	515	17.80	**1.62**	**1.26-2.07**
541 Other surveillance and security workers	402	13.30	1.21	0.92-1.59
5 Combined data for service, care and shop sales workers equivalent lacking 3-digit information or power	*315*	*17.34*	***1.58***	***1.16-2.16***
**611Market gardeners and crop growers**	394	17.49	**1.60**	**1.21-2.11**
612 Animal breeders and keepers	216	13.36	1.22	0.84-1.76
613 Mixed crop and animal breeders	144	13.66	1.25	0.79-1.97
621 Forestry and related workers	58	12.07	1.11	0.54-2.28
6 Combined data for agricultural, horticultural, forestry and fishery workers lacking 3-digit information or power	*49*	*13.00*	*1.17*	*0.54-2.55*
**711 Carpenters, bricklayers and construction workers**	1 472	17.96	**1.62**	**1.39-2.64**
**712 Roofers, floor layers, plumbers and pipefitters**	716	16.71	**1.51**	**1.22-1.86**
**713 Painters, Lacquerers, Chimney-sweepers and related trades workers**	494	18.04	**1.63**	**1.27-2.09**
**721 Sheet and structural metal workers, moulders and welders, and related workers**	812	21.65	**1.95**	**1.60-2.39**
**722 Blacksmiths, toolmakers and related trades workers**	839	14.91	**1.35**	**1.10-1.64**
**723 Machinery mechanics and fitters**	956	14.10	**1.28**	**1.06-1.54**
731 Precision-instrument makers and handicraft workers	20	11.10	0.96	0.29-3.25
732 Printing trades workers	149	12.69	1.15	0.73-1.80
741 Electrical equipment installers and repairers	552	11.28	1.02	0.81-1.30
742 Electronics and telecommunications installers and repairers	141	10.27	1.93	0.59-1.48
**752 Wood treaters, cabinet-makers and related trades workers**	956	18.24	**1.65**	**1.37-1.99**
**761 Butchers, bakers and food processors**	57	27.76	**2.58**	**1.25-5.33**
7 Combined data for building and manufacturing workers lacking 3-digit information or power	*190*	*19.51*	***1.77***	***1.19-2.64***
**811 Mining and mineral processing plant operators**	153	17.78	**1.61**	**1.03-2.50**
**812 Metal processing and finishing plant operators**	390	18.39	**1.66**	**1.25-2.20**
813 Machine operators, chemical and pharmaceutical products	23	12.92	1.20	0.39-3.74
**814 Machine operators, rubber, plastic and paper products**	266	17.54	**1.58**	**1.13-2.22**
**816 Machine operators, food and related products**	344	25.77	**2.33**	**1.73-3.15**
817 Wood processing and papermaking plant operators			1.06	0.76-1.49
819 Process control technicians	231	10.16	0.92	0.64-1.32
**821 Assemblers**	1 061	16.47	**1.49**	**1.25-1.78**
831 Train operators and related workers	15	5.56	0.54	0.13-2.21
**832 Car, van and motorcycle drivers**	267	16.19	**1.47**	**1.05-2.07**
**833 Heavy truck and bus drivers**	1 019	15.80	**1.41**	**1.20-1.65**
834 Mobile plant operators	527	12.28	1.11	0.87-1.42
8 Combined data for mechanical manufacturing and transport workers, etc. lacking 3-digit information or power	*240*	*15.57*	*1.40*	*0.98-1.99*
**911 Cleaners and helpers**	240	25.25	**2.30**	**1.61-3.28**
**932 Manufacturing labourers**	422	22.37	**2.03**	**1.55-2.66**
**933 Dockers and ground personnel**	231	17.71	**1.61**	**1.12-2.31**
**941 Fast-food workers, food preparation assistants**	165	18.88	**1.73**	**1.12-2.65**
**961 Recycling collectors**	210	20.71	**1.88**	**1.29-2.75**
**962 Newspaper distributors, janitors and other service workers**	633	21.12	**1.92**	**1.54-2.41**
9 Combined data for elementary occupations lacking 3-digit information or power	*218*	*22.06*	***2.00***	***1.38-2.90***
**Not in the labour force**	7 516	29.97	**2.74**	**2.51-2.99**

**Unit occupational groups** (4-digit level)	**Events (N)**	**Events (N) per 10 000 person-years**	**IRR**	**95% CI**
0110 Commissioned armed forces officers	*147*	*12.28*	*1.15*	*0.74-1.77*
0 Combined data for armed forces occupations lacking 4-digit information or power	*8*	*8.41*	*0.79*	*0.12-5.00*
**1120 Directors and chief executives**	151	6.20	**0.58**	**0.38-0.89**
1211 Finance managers, level 1	40	5.77	0.54	0.23-1.23
1230 Administration and planning managers	19	4.83	0.45	0.14-1.51
**1251 Sales and marketing managers, level 1**	66	4.67	**0.43**	**0.23-0.82**
1252 Sales and marketing managers, level 2	22	5.82	0.52	0.17-1.59

1291 Administration and service managers not elsewhere classified. level 1	64	5.79	0.54	0.28-1.03
1362 Production managers in construction and mining, level 2	108	7.39	0.68	0.41-1.14
1372 Production managers in manufacturing, level 2	50	5.38	0.50	0.24-1.05
1591 Managers in public services not elsewhere classified, level 1	46	6.17	0.57	0.26-1.24
1592 Operations managers in public services not elsewhere classified, level 2	50	5.69	0.53	0.25-1.11
1612 Financial and insurance managers, level 2	66	8.40	0.78	0.41-1.49
1711 Hotel and conference managers, level 1	34	8.68	0.77	0.31-1.90
1722 Restaurant managers, level 2	175	9.79	0.91	0.61-1.36
1792 Other services managers not elsewhere classified, level 2	50	7.84	0.73	0.35-1.53
1 Combined data for managers lacking 4-digit information or power	*491*	*5.61*	***0.52***	***0.41-0.67***
2142 Engineering professionals in building construction	65	6.28	0.58	0.30-1.12
**2143 Engineering professionals in electrical, electronics and telecommunications**	109	4.78	**0.44**	**0.27-0.73**
**2144 Engineering professionals in mechanical technology**	81	4.91	**0.46**	**0.25-0.82**
**2149 Engineering professionals not elsewhere classified**	67	4.55	**0.42**	**0.22-0.80**
2211 Specialist physicians	105	7.06	0.66	0.40-1.11
2311 Professors	117	7.92	0.74	0.46-1.21
2320 Vocational education teachers	64	12.06	1.14	0.59-2.20
2330 Secondary education teachers	106	7.63	0.71	0.43-1.19
2341 Primary school teachers	334	9.68	0.90	0.68-1.21
2343 Preschool teachers	42	11.57	1.07	0.48-2.41
2359 Teaching professionals not elsewhere classified	27	7.12	0.67	0.25-1.85
2411 Accountants	75	6.72	0.63	0.34-1.15
2419 Economists not elsewhere classified	74	6.65	0.62	0.34-1.14
**2421 Management and organisation analysts**	55	4.70	**0.44**	**0.22-0.89**
2422 Policy administration professionals	209	7.48	0.70	0.49-1.01
2431 Advertising and marketing professionals	79	6.61	0.61	0.34-1.11
**2511 System analysts and ICT-architects**	101	5.92	**0.55**	**0.33-0.93**
**2512 Software- and system developers**	431	5.95	**0.55**	**0.43-0.71**
2642 Journalists and related professionals	91	7.20	0.67	0.39-1.17
2 Combined data for occupations requiring advanced level of higher education lacking 4-digit information or power	*1 131*	*7.60*	***0.71***	***0.60-0.84***
**3112 Construction and manufacturing engineering technicians**	160	6.98	**0.65**	**0.43-0.99**
**3113 Electronics engineering technicians**	179	6.17	**0.57**	**0.39-0.85**
3114 Mechanical engineering technicians	194	7.42	0.69	0.47-1.01
**3119 Technicians not elsewhere classified**	165	6.38	**0.59**	**0.39-0.90**
3121 Construction and mining supervisors	10	5.81	0.52	0.10-2.71
3312 Bank clerk	77	6.91	0.65	0.36-1.18
3313 Accounting associate professionals	17	4.88	0.45	0.13-1.60
3321 Insurance sellers and insurance advisers	56	7.39	0.69	0.34-1.39
**3322 Commercial sales representatives**	604	7.27	**0.67**	**0.54-0.84**
**3323 Buyers and purchasers**	61	7.34	**0.47**	**0.24-0.93**
3334 Real estate agents	25	6.65	0.63	0.22-1.79
3339 Business services agents not elsewhere classified	134	9.45	0.88	0.56-1.39
3360 Police Officers	148	9.63	0.90	0.59-1.40
**3411 Social work associate professionals**	146	17.92	**1.67**	**1.08-2.58**
3423 Recreation and related associate professionals	54	12.45	1.16	0.57-2.38
3511 ICT operations technicians	13	7.55	0.73	0.17-3.10
3514 Computer network and systems technicians	266	8.71	0.81	0.58-1.12
3 Combined data for occupations requiring higher education qualifications or equivalent lacking 4-digit information or power	*1 161*	*8.33*	***0.78***	***0.66-0.91***
4111 Economic assistants	20	10.45	1.01	0.31-3.24
4119 Office clerks not elsewhere classified	317	10.47	0.98	0.73-1.32
**4322 Warehouse and terminal Staff**	660	13.55	**1.26**	**1.02-1.56**
4323 Transport coordinators	15	7.45	0.66	0.17-2.56
4420 Postmen and postal facility workers	167	11.35	1.01	0.71-1.61
4 Combined data for administration and customer service clerks lacking 4-digit information or power	*516*	*12.30*	*1.15*	*0.91-1.46*
**5120 Cooks and cold-buffet managers**	219	19.11	**1.77**	**1.23-2.53**
5131 Waiters	45	14.66	1.41	0.64-3.08
**5152 Building caretakers**	951	14.26	**1.34**	**1.07-1.67**
5221 Shopkeepers and shop supervisors, selling in stores	81	11.30	1.04	0.58-1.86
5222 Shop sales, groceries	246	13.65	1.28	0.91-1.79

5223 Shop sales, specialty stores	492	11.55	1.08	0.85-1.37
5241 Event salespeople and shop demonstrators	45	22.40	2.15	0.98-4.71
5311 Child care workers	109	14.24	1.34	0.81-2.2
**5321 Assistant nurses, home care and homes for the elderly**	578	26.58	**2.49**	**1.99-3.12**
**5323 Assistant nurses, hospital ward**	226	22.01	**2.05**	**1.44-2.92**
**5342 Personal care providers**	351	18.55	**1.73**	**1.30-2.31**
5411 Firefighters	66	10.55	0.99	0.52-1.91
5412 Prison guards	22	10.45	1.00	0.33-3.07
5413 Security guards	154	14.37	1.33	0.87-2.03
5 Combined data for service, care and shop sales workers equivalent lacking 4-digit information or power	*959*	*16.06*	***1.50***	***1.26-1.80***
6111 Field crop and vegetable growers	65	11.10	1.06	0.55-2.03
**6113 Gardeners, parks and grounds**	253	21.28	**1.99**	**1.43-2.78**
6121 Livestock and dairy producers	159	12.21	1.14	0.75-1.74
6130 Mixed crop and animal breeders	121	13.79	1.29	0.80-2.08
6210 Forestry and related workers	56	11.78	1.12	0.55-2.26
6 Combined data for agricultural, horticultural, forestry and fishery workers lacking 4-digit information or power	*207*	*15.33*	*1.44*	*0.99-2.08*
7111 Woodworkers, carpenters	184	11.06	1.03	0.69-1.51
**7112 Bricklayers and related workers**	140	18.65	**1.70**	**1.09-2.66**
**7113 Concrete placers, concrete finishers and related workers**	179	19.19	**1.78**	**1.20-2.64**
7114 Rail and road construction workers	246	14.45	1.35	0.96-1.89
**7119 Building frame and related trades workers not elsewhere classified**	450	23.30	**2.17**	**1.68-2.79**
7125 Plumbing and central heating fitters	299	14.44	1.34	0.99-1.82
7131 Painters and related workers	245	14.75	1.37	0.98-1.92
**7212 Welders and flame cutters**	397	22.35	**2.08**	**1.59-2.72**
**7214 Sheet metal workers**	195	19.83	**1.84**	**1.26-2.68**
**7223 Machine-tool operators**	646	14.64	**1.36**	**1.10-1.68**
7231 Vehicle mechanics and repairers	420	12.92	1.20	0.93-1.56
7233 Agricultural and industrial machinery mechanics and repairers	380	13.60	1.27	0.96-1.67
7322 Printers	25	11.26	1.00	0.35-2.86
7411 Electricians, installation and service	294	10.37	0.97	0.71-1.32
7412 Electrical mechanics and fitters	168	12.21	1.14	0.76-1.71
7420 Electronics repairers and telecom electricians	141	10.27	0.96	0.61-1.49
**7522 Cabinet-makers and related workers**	736	18.46	**1.72**	**1.41-2.10**
**7523 Machine operator, Wood-products**	219	17.70	**1.65**	**1.15-2.36**
7 Combined data for building and manufacturing workers lacking 4-digit information or power	*1 990*	*19.02*	***1.77***	***1.56-2.02***
8142 Machine operators, plastic products	70	18.17	1.68	0.89-3.15
8172 Papermaking plant operators	63	10.08	0.93	0.48-1.80
8173 Wood processing, sawmill and plywood plant operators	72	11.53	1.07	0.57-1.98
8191 Power production and water treatment plant operators	78	9.78	0.91	0.50-1.65
8192 Chemical processing plant controllers	41	10.02	0.89	0.39-2.03
8211 Mechanical machinery assemblers	188	15.37	1.43	0.97-2.10
8212 Electrical and electronic equipment assemblers	102	13.38	1.23	0.73-2.07
**8213 Metal-, rubber- and plastic-products assemblers**	241	17.89	**1.66**	**1.18-2.34**
**8219 Assemblers not elsewhere classified**	317	16.35	**1.52**	**1.13-2.05**
**8321 Taxi, car, and van drivers**	251	17.14	**1.61**	**1.15-2.25**
8331 Bus and tram drivers	186	11.89	1.12	0.76-1.65
**8332 Heavy truck and lorry drivers**	942	14.79	**1.38**	**1.15-1.65**
8341 Forestry and agricultural machinery operators	77	8.87	0.83	0.45-1.51
8342 Heavy Equipment Operators	233	12.22	1.14	0.81-1.61
8344 Fork-lift drivers	137	13.56	1.26	0.81-1.98
8 Combined data for mechanical manufacturing and transport workers, etc. lacking 4-digit information or power	*2 176*	*17.49*	***1.64***	***1.44-1.85***
**9111 Cleaners and helpers in offices, hotels and other establishments**	105	23.24	**2.16**	**1.29-3.61**
**9320 Manufacturing labourers**	371	20.78	**1.94**	**1.47-2.56**
**9332 Ground personnel, movers and stockers**	200	17.25	**1.61**	**1.11-2.35**
**9412 Restaurant and kitchen helpers**	134	17.80	**1.68**	**1.06-2.65**
**9610 Recycling collectors**	199	20.11	**1.88**	**1.29-2.74**
9622 Janitors and related workers	78	14.83	1.41	0.78-2.55
**9629 Other service workers not elsewhere classified**	400	24.57	**2.30**	**1.76-3.01**
9 Combined data for elementary occupations lacking 4-digit information or power	*632*	*23.22*	***2.17***	***1.75-2.69***

**Not in the labour force**	7 516	29.97	**2.82**	**2.60-3.06**

Note: bold letters indicate significant risk estimates.
Occupational groups on 1-digit level; 10 of 10 (100%), on 2-digit level; 42 of 46 (91.3%), on 3-digit level; 94 of 148 (63.5%), on 4-digit level; 115 of 429 (26.8%) included enough person-years to be used in the analyses.
