# Supplementary material for: Occupational groups and risk of suicidal behavior in men: a Swedish national cohort study during 2002–2019
Source: BMC Public Health. 2024 Dec 18;24:3515. doi: 10.1186/s12889-024-20887-x (PMC11657517; doi:10.1186/s12889-024-20887-x)
Supplement: Supplementary file 3 — Additional file 3. Risk for suicidal behaviour among men without psychiatric disorders at or before conscription. Incidence rate ratios (IRR) with 95% confidence intervals for suicidal behaviour (fatal or non-fatal) in analyses where men with psychiatric disorders at conscription (age 16–25) were excluded (light grey bars), or not (dark grey bars). In (a) IRRs lower or higher than the incidence rate of the total study population for the major occupational groups (1-digit level) are presented In (a) and IRRs higher than the incidence rate of the total study population for the sub-major occupational groups (2-digit level) are shown [file 12889_2024_20887_MOESM3_ESM.pdf]

Additional file 3 Nyberg et al., 2024. Occupational groups and risk of suicidal behavior in men: a Swedish national cohort study during 2002-2019.

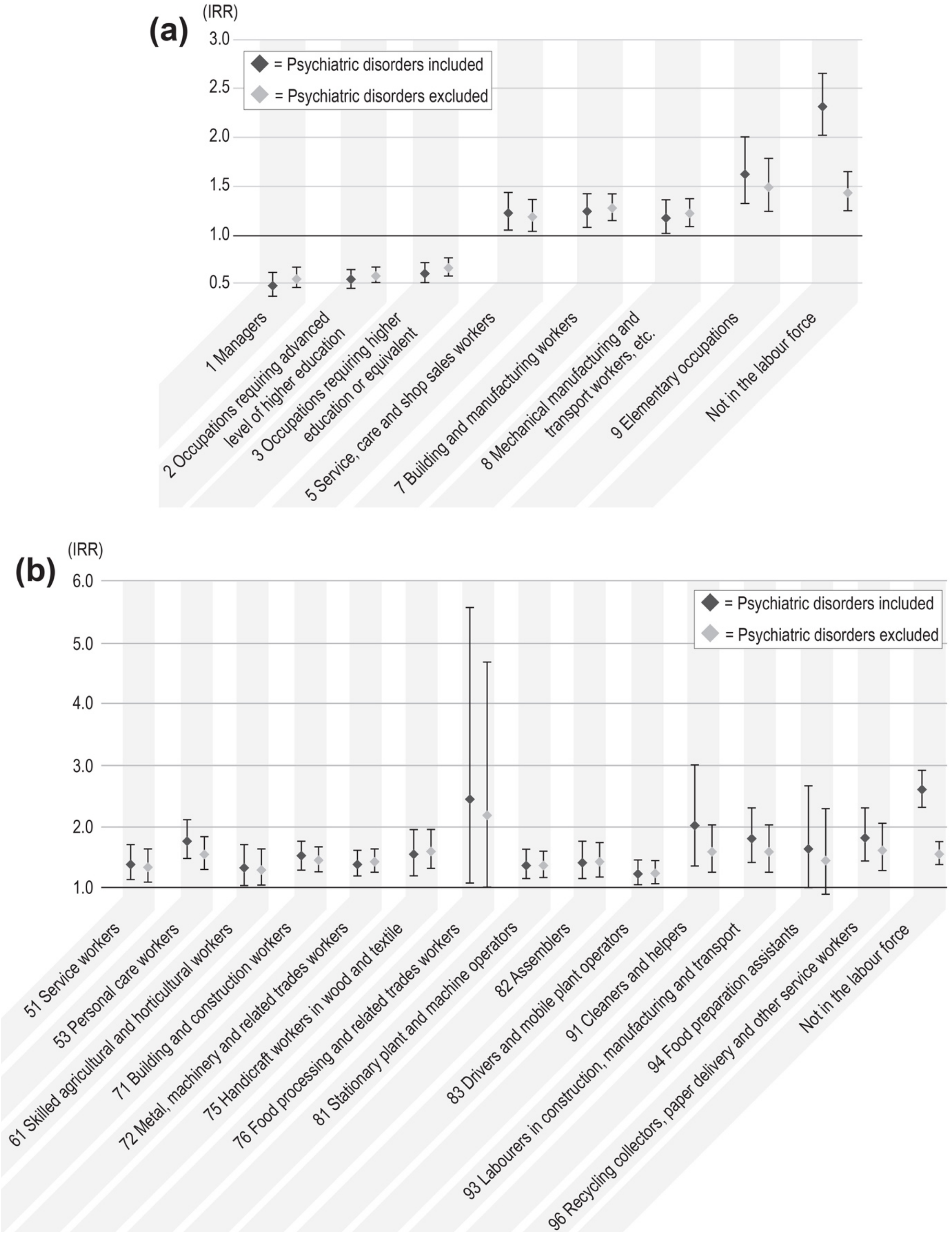

Additional file 3. Risk for suicidal behaviour among men without psychiatric disorders at or before conscription. Incidence rate ratios (IRR) with 95% confidence intervals for suicidal behaviour (fatal or non-fatal) in analyses where men with psychiatric disorders at conscription (age 16-25) were excluded (light grey bars), or not (dark grey bars). In (a) IRRs lower or higher than the incidence rate of the total study population for the major occupational groups (1-digit level) are presented In (a) and IRRs higher than the incidence rate of the total study population for the sub-major occupational groups (2-digit level) are shown.
