# Supplementary material for: Occupational groups and risk of suicidal behavior in men: a Swedish national cohort study during 2002–2019
Source: BMC Public Health. 2024 Dec 18;24:3515. doi: 10.1186/s12889-024-20887-x (PMC11657517; doi:10.1186/s12889-024-20887-x)
Supplement: Supplementary file 4 — Additional file 4. Non-significant risk estimates for non-fatal self-harm among occupational groups. Number of events, events per 10 000 person-years, incidence rate ratios (IRR) and 95% confidence intervals (CI) for non-fatal self-harm calculated separately for 1- to 4-digit level occupational groups compared to the incidence rate of the total study population. Only groups with sufficient sample size are shown [file 12889_2024_20887_MOESM4_ESM.pdf]

Additional file 4. Non-significant risk estimates for non-fatal self-harm among occupational groups. Number of events, events per 10 000 person-years, incidence rate ratios (IRR) and 95% confidence intervals (CI) for non-fatal self-harm calculated separately for 1- to 4-digit level occupational groups compared to the incidence rate of the total study population. Only groups with sufficient sample size are shown.

**Major occupational groups** (1-digit level)	**Events (N)**	**Events (N) per 10 000 person-years**	**IRR**	**95% CI**
0 Armed forces occupations	136	10.52	0.94	0.46-1.92
4 Administration and customer service clerks	1 437	10.29	0.92	0.73-1.17
6 Agricultural, horticultural, forestry and fishery workers	695	12.02	1.11	0.80-1.53
**Sub-major occupational groups** (2-digit level)[a]	**Events (N)**	**Events (N) per 10 000 person-years**	**IRR**	**95% CI**
01 Officers	132	10.97	1.13	0.66-1.92
0 Compressed data for armed forces occupations lacking 2-digit information	*4*	*4.50*	*0.46*	*0.02-9.12*
14 Education managers	14	6.81	0.82	0.16-4.09
16 Financial and insurance services branch managers	50	6.11	0.64	0.27-1.51
17 Hotel, restaurant, retail and other services managers	273	7.36	0.76	0.52-1.11
1 Combined data for managers lacking 2-digit information or power	*16*	*3.26*	*0.33*	*0.07-1.46*
22 Occupations requiring advanced academic competence in health care	263	6.59	0.69	0.47-1.01
23 Occupations requiring advanced academic competence in education	738	7.80	0.81	0.63-1.03
32 Occupations requiring higher education qualification or the equivalent in healthcare and laboratory	74	8.06	0.83	0.41-1.69
34 Occupations requiring higher education qualification or the equivalent in culture, wellness and social work	385	10.81	1.11	0.80-1.53
41 Office clerks	363	8.29	0.85	0.61-1.19
42 Customer services clerk	108	10.04	1.00	0.55-1.79
43 Stock clerks	791	11.90	1.22	0.96-1.55
44 Other office and service workers	164	9.66	1.01	0.63-1.63
4 Combined data for administration and customer service clerks lacking 2-digit information or power	*11*	*6.78*	*1.82*	*0.13-4.99*
52 Sales workers	949	10.77	1.09	0.88-1.36
54 Surveillance workers and fire fighters	353	11.68	1.19	0.85-1.67
62 Skilled forestry and fishery workers	45	7.84	0.87	0.35-2.13
6 Combined data for agricultural, horticultural, forestry and fishery workers lacking 3-digit information or power	*29*	*10.23*	*1.00*	*0.33-3.06*
73 Precision-instrument makers, printing and handicraft workers	190	10.82	1.12	0.72-1.75
74 Electrical and electronic trades workers	605	9.65	0.99	0.76-1.30
7 Combined data for building and manufacturing workers lacking 2-digit information or power	*101*	*24.21*	*2.47*	*1.35-4.53*
9 Combined data for elementary occupations lacking 2-digit information or power	*86*	*19.16*	*2.01*	*1.05-3.88*
**Minor occupational groups** (3-digit level)[a]	**Events (N)**	**Events (N) per 10 000 person-years**	**IRR**	**95% CI**
011 Commissioned armed forces officers	132	10.97	1.17	0.73-1.86
0 Compressed data for armed forces occupations lacking 3-digit information	*4*	*4.50*	*0.47*	*0.03-6.69*
121 Finance managers	40	4.66	0.50	0.22-1.17
123 Administration and planning managers	17	4.32	0.49	0.13-1.76
131 Information and communications technology service managers	23	5.05	0.51	0.17-1.55
132 Supply, logistics and transport managers	18	3.97	0.40	0.12-1.41
136 Production managers in construction and mining	102	6.27	0.67	0.39-1.13
161 Financial and insurance managers	50	6.11	0.66	0.31-1.40
171 Hotel and conference managers	31	7.75	0.79	0.30-2.04
172 Restaurant managers	155	8.50	0.92	0.59-1.41
179 Other services managers not elsewhere classified	40	5.74	0.62	0.28-1.44
1 Combined data for managers lacking 3-digit information or power	*210*	*4.39*	*0.47*	*0.32-0.68*
217 Designers	31	6.52	0.66	0.26-1.72
222 Nursing professionals	39	9.29	0.94	0.40-2.21
231 University and higher education teachers	123	6.46	0.69	0.42-1.11
232 Vocational education teachers	54	10.17	1.15	0.56-2.39
233 Secondary education teachers	88	6.18	0.66	0.37-1.17
234 Primary- and pre-school teachers	381	8.82	0.94	0.71-1.24

235 Teaching professionals not elsewhere classified	78	7.46	0.81	0.44-1.47
243 Marketing and public relations professionals	77	5.35	0.56	0.31-1.03
261 Legal professionals	56	4.94	0.53	0.26-1.07
264 Authors, journalists and linguists	75	5.50	0.58	0.32-1.08
265 Social work and counselling professionals	69	7.70	0.82	0.43-1.55
2 Combined data for occupations requiring advanced level of higher education lacking 3-digit information or power	*381*	*6.86*	*0.74*	*0.56-0.98*
312 Construction and manufacturing supervisors	43	6.94	0.56	0.25-1.25
315 Ship and aircraft controllers and technicians	43	6.94	0.75	0.33-1.68
321 Medical and pharmaceutical technicians	69	7.88	0.84	0.44-1.60
333 Business services agents	231	7.38	0.79	0.55-1.12
335 Tax and related government associate professionals	109	7.25	0.78	0.47-1.30
336 Police officers	130	8.46	0.91	0.57-1.45
341 Social work and religious associate professionals	137	14.35	1.53	0.97-2.41
342 Athletes, fitness instructors and recreational workers	101	10.47	1.07	0.63-1.83
343 Photographers, interior decorators and entertainers	64	8.61	0.88	0.45-1.71
351 ICT operations and user support technicians	325	7.08	0.74	0.55-1.01
3 Combined data for occupations requiring higher education qualifications or equivalent lacking 3-digit information or power	*131*	*7.31*	*0.80*	*0.50-1.28*
411 Office assistants and other secretaries	363	8.29	0.88	0.66-1.18
422 Client information clerks	102	10.24	1.05	0.62-1.79
442 Postmen and postal facility workers	152	9.90	1.07	0.69-1.66
4 Combined data for administration and customer service clerks lacking 3-digit information or power	*29*	*7.14*	*0.80*	*0.30-2.16*
513 Waiters and bartenders	44	13.54	1.41	0.63-3.15
522 Shop staff	826	10.38	1.09	0.90-1.33
523 Cashiers and related clerks	22	11.27	1.17	0.38-3.65
524 Event seller and telemarketers	46	19.63	1.17	0.38-3.65
531 Child care workers and teachers aides	146	12.83	1.32	0.85-2.05
541 Other surveillance and security workers	353	11.68	1.23	0.92-1.65
5 Combined data for service, care and shop sales workers equivalent lacking 3-digit information or power	*268*	*14.75*	*1.60*	*1.15-2.22*
612 Animal breeders and keepers	174	10.77	1.18	0.79-1.77
613 Mixed crop and animal breeders	121	11.47	1.25	0.77-2.04
621 Forestry and related workers	36	7.49	0.85	0.35-2.07
6 Combined data for agricultural, horticultural, forestry and fishery workers lacking 3-digit information or power	*38*	*10.08*	*1.05*	*0.44-2.48*
731 Precision-instrument makers and handicraft workers	19	10.55	1.10	0.32-3.71
732 Printing trades workers	129	11.00	1.17	0.73-1.88
741 Electrical equipment installers and repairers	480	9.81	1.04	0.81-1.34
742 Electronics and telecommunications installers and repairers	125	9.11	0.97	0.60-1.57
7 Combined data for building and manufacturing workers lacking 3-digit information or power	*162*	*16.64*	*1.78*	*1.17-2.72*
813 Machine operators, chemical and pharmaceutical products	21	11.80	1.23	0.39-2.29
817 Wood processing and papermaking plant operators	219	9.57	1.02	0.15-1.47
819 Process control technicians	186	8.18	0.88	0.59-1.30
831 Train operators and related workers	12	4.45	0.55	0.12-2.55
834 Mobile plant operators	438	10.21	1.09	0.84-1.41
8 Combined data for mechanical manufacturing and transport workers, etc. lacking 3-digit information or power	*204*	*15.57*	*1.39*	*0.96-2.03*
9 Combined data for elementary occupations lacking 3-digit information or power	*188*	*19.03*	*2.04*	*1.38-3.01*

**Unit occupational groups** (4-digit level) [a]	**Events (N)**	**Events (N) per 10 000 person-years**	**IRR**	**95% CI**
0110 Commissioned armed forces officers	132	11.03	1.22	0.78-1.91
0 Combined data for armed forces occupations lacking 4-digit information or power	*4*	*4.20*	*0.46*	*0.04-5.90*
1211 Finance managers, level 1	36	5.19	0.58	0.25-3.37
1230 Administration and planning managers	17	4.32	0.50	0.15-1.74
1252 Sales and marketing managers, level 2	19	5.02	0.53	0.16-1.70
1291 Administration and service managers not elsewhere classified, level 1	56	5.07	0.56	0.28-1.12
1362 Production managers in construction and mining, level 2	96	6.56	0.72	0.43-1.22
1372 Production managers in manufacturing, level 2	39	4.20	0.47	0.21-1.06
1591 Managers in public services not elsewhere classified, level 1	39	5.23	0.59	0.26-1.34
1592 Operations managers in public services not elsewhere classified, level 2	44	5.01	0.56	0.26-1.21

1612 Financial and insurance managers, level 2	49	6.24	0.70	0.34-1.46
1711 Hotel and conference managers, level 1	31	7.91	0.83	0.33-2.08
1722 Restaurant managers, level 2	154	8.61	0.96	0.63-1.46
1792 Other services managers not elsewhere classified, level 2	38	5.96	0.67	0.29-1.53
1 Combined data for managers lacking 4-digit information or power	*417*	*4.77*	*0.53*	*0.41-0.68*
2142 Engineering professionals in building construction	53	5.12	0.56	0.28-1.14
2211 Specialist physicians	76	5.11	0.58	0.32-1.04
2311 Professors	100	6.77	0.75	0.45-1.25
2320 Vocational education teachers	54	10.17	1.19	0.59-2.40
2330 Secondary education teachers	88	6.33	0.70	0.41-1.22
2341 Primary school teachers	292	8.46	0.93	0.69-1.27
2343 Preschool teachers	39	10.74	1.13	0.50-2.57
2359 Teaching professionals not elsewhere classified	18	4.75	0.56	0.17-1.86
2411 Accountants	68	6.10	0.68	0.36-1.26
2419 Economists not elsewhere classified	65	5.84	0.65	0.34-1.22
2422 Policy administration professionals	180	6.44	0.72	0.49-1.06
2431 Advertising and marketing professionals	70	5.86	0.64	0.35-1.18
2642 Journalists and related professionals	72	5.70	0.63	0.34-1.15
2 Combined data for occupations requiring advanced level of higher education lacking 4-digit information or power	*946*	*6.35*	*0.70*	*0.59-0.84*
3112 Construction and manufacturing engineering technicians	142	6.19	0.68	0.44-1.05
3114 Mechanical engineering technicians	171	6.54	0.72	0.48-1.07
3121 Construction and mining supervisors	9	5.23	0.56	0.10-3.09
3312 Bank clerk	64	5.75	0.63	0.33-1.20
3313 Accounting associate professionals	14	4.02	0.43	0.11-1.70
3321 Insurance sellers and insurance advisers	50	6.59	0.73	0.35-1.51
3334 Real estate agents	20	5.32	0.62	0.20-1.96
3339 Business services agents not elsewhere classified	126	8.88	0.98	0.62-1.55
3360 Police Officers	130	8.46	0.94	0.60-1.48
3423 Recreation and related associate professionals	49	11.30	1.20	0.57-2.49
3511 ICT operations technicians	11	6.38	0.69	0.15-3.24
3514 Computer network and systems technicians	231	7.57	0.82	0.58-1.16
3 Combined data for occupations requiring higher education qualifications or equivalent lacking 4-digit information or power	*998*	*7.16*	*0.79*	*0.66-0.94*
4111 Economic assistants	18	9.40	1.02	0.30-3.41
4119 Office clerks not elsewhere classified	265	8.75	0.97	0.70-1.33
4323 Transport coordinators	12	5.96	0.64	0.15-2.80
4420 Postmen and postal facility workers	142	9.65	1.08	0.70-1.67
4 Combined data for administration and customer service clerks lacking 4-digit information or power	*425*	*10.13*	*1.11*	*0.86-1.44*
5131 Waiters	40	13.03	1.41	0.63-3.18
5221 Shopkeepers and shop supervisors, selling in stores	67	9.34	0.99	0.53-1.86
5222 Shop sales, groceries	215	11.93	1.30	0.91-1.85
5223 Shop sales, specialty stores	416	9.77	1.07	0.82-1.38
5241 Event salespeople and shop demonstrators	39	19.41	2.10	0.92-4.79
5311 Child care workers	95	12.41	1.32	0.78-2.24
5411 Firefighters	57	9.11	1.04	0.52-2.05
5412 Prison guards	19	9.02	0.98	0.30-3.17
5413 Security guards	141	13.15	1.40	0.91-2.16
5 Combined data for service, care and shop sales workers equivalent lacking 4-digit information or power	*792*	*13.27*	*1.47*	*1.21-1.78*
6111 Field crop and vegetable growers	51	8.71	1.03	0.50-2.11
6121 Livestock and dairy producers	126	9.68	1.11	0.70-1.75
6130 Mixed crop and animal breeders	100	11.39	1.30	0.77-2.17
6210 Forestry and related workers	34	7.15	0.84	0.35-2.03
6 Combined data for agricultural, horticultural, forestry and fishery workers lacking 4-digit information or power	*177*	*13.11*	*1.42*	*0.96-2.10*
7111 Woodworkers, carpenters	161	9.68	1.06	0.70-1.59
7114 Rail and road construction workers	199	11.70	1.30	0.90-1.88
7131 Painters and related workers	208	12.52	1.38	0.96-1.97
7231 Vehicle mechanics and repairers	371	11.41	1.26	0.96-1.65
7233 Agricultural and industrial machinery mechanics and repairers	321	11.48	1.27	0.95-1.71
7322 Printers	22	9.91	1.06	0.36-3.17

7411 Electricians, installation and service	262	9.25	1.02	0.74-1.40
7412 Electrical mechanics and fitters	142	10.32	1.14	0.74-1.76
7420 Electronics repairers and telecom electricians	125	9.11	1.01	0.63-1.59
7 Combined data for building and manufacturing workers lacking 4-digit information or power	*1 721*	*16.45*	*1.82*	*1.58-2.08*
8142 Machine operators, plastic products	61	15.83	1.70	0.88-3.29
8172 Papermaking plant operators	56	8.96	0.99	0.50-1.98
8173 Wood processing, sawmill and plywood plant operators	56	8.97	1.00	0.50-1.98
8191 Power production and water treatment plant operators	63	7.90	0.89	0.47-1.71
8192 Chemical processing plant controllers	33	8.06	0.85	0.35-2.07
8211 Mechanical machinery assemblers	158	12.92	1.41	0.93-2.12
8212 Electrical and electronic equipment assemblers	82	10.76	1.14	0.65-2.01
8331 Bus and tram drivers	153	9.78	1.11	0.73-1.69
8341 Forestry and agricultural machinery operators	62	7.14	0.79	0.41-1.51
8342 Heavy Equipment Operators	195	10.23	1.13	0.78-1.64
8344 Fork-lift drivers	119	11.78	1.30	0.81-2.09
8 Combined data for mechanical manufacturing and transport workers, etc. lacking 4-digit information or power	*1 855*	*14.91*	*1.65*	*1.44-1.88*
9622 Janitors and related workers	63	11.98	1.41	0.74-2.70
9 Combined data for elementary occupations lacking 4-digit information or power	*545*	*20.02*	*2.21*	*2.76-2.77*

[a] Occupational 2- to 4-digit codes with too few person-years to analyse are not presented in the table due to space. However, these small occupational 2- to 4-digit groups are included in the analyses combined with occupational codes with missing information. All occupational groups on 1-digit level, 42 of 46 (91.3%) groups on 2-digit level, 92 of 148 (62.2%) groups on 3-digit level and 115 of 429 (26.8%) groups on 4-digit level included enough person-years to be used in the analyses.
Bold letters indicate significant risk estimates.
